# NIPBL-mediated RAD21 facilitates tumorigenicity by the PI3K pathway in non-small-cell lung cancer

**DOI:** 10.1038/s42003-024-05801-w

**Published:** 2024-02-20

**Authors:** Xiaoling Xu, Ding Wang, Weizhen Xu, Huihui Li, Ning Chen, Na Li, Qifeng Yao, Wei Chen, Jianxiang Zhong, Weimin Mao

**Affiliations:** 1 Department of Radiation Oncology, Shanghai Pulmonary Hospital, Tongji University School of Medicine, 507 Zhengmin Road, Shanghai, China; 2 Key laboratory on Diagnosis and Treatment Technology on Thoracic Cancer, Zhejiang Cancer Research Institute, 38 Guangji Road, Hangzhou, China; 3 The Second Clinical Medical College of Wenzhou Medical University, 268 West Xueyue Road, Wenzhou, China; 4 The Second Clinical Medical College of Zhejiang Chinese Medical University, 548 Binwen Road, Hangzhou, China; 5 School of Life Science and Technology, Southeast University, 2 Sipailou, Nanjing, China; 6 Department of Thoracic Oncology, Zhejiang Cancer Hospital, Institute of Basic Medicine and Cancer (IBMC), Chinese Academy of Sciences, 1 Banshan East Road, Hangzhou, China

**Keywords:** Non-small-cell lung cancer, DNA methylation

## Abstract

It is urgent to identify novel early diagnostic markers and therapeutic targets for non-small-cell lung cancer (NSCLC), which accounts for 85% of lung cancer cases and has a 5-year survival rate of 4–17%. Here, chromatin immunoprecipitation (ChIP) was used to identify DNA‒protein interactions, RNA methylation was determined by methylated RNA immunoprecipitation (MeRIP), RNA stability was tested by an RNA decay assay. We showed that RAD21, a member of the cohesin complex, is upregulated in NSCLC tissues and cell lines and found to be an independent prognostic factor for overall survival (OS) of NSCLC patients. Mechanistically, the cohesin loading factor Nipped-B-Like Protein (NIPBL) promoted *RAD21* gene transcription by enhancing histone H3 lysine 27 (H3K27) demethylation via recruiting lysine demethylase 6B (KDM6B) to the *RAD21* gene promoter. RAD21 enhanced phosphatidylinositol 3-kinase (PI3K) gene transcription, and NIPBL reversed the effect of enhancer of zeste 2; catalytic subunit of polycomb repressive complex 2 (EZH2) on RAD21-mediated *PI3K* gene transcription by disrupting the association between EZH2 and RAD21. Moreover, NIPBL level was increased by stabilization of its transcripts through mRNA methylation. These findings highlight the oncogenic role of RAD21 in NSCLC and suggest its use as a potential diagnostic marker and therapeutic target for NSCLC.

## Abstract (web summary)

The oncogenic role of RAD21 in NSCLC is mediated by NIPBL via H3K27 demethylation and suggest its use as a potential novel diagnostic marker and therapeutic target for NSCLC.

## Introduction

Lung cancer is the most commonly diagnosed cancer and the leading cause of cancer-related deaths in the world^1–3^. More than 2 million new cases of lung cancer are diagnosed every year, with approximately 1.6 million deaths attributed to this disease worldwide^3,4^. Among the histological types, non-small-cell lung cancer (NSCLC) accounts for 85% of all lung cancer cases diagnosed^5^. Numerous technical and pharmacological advances have been made to improve the prognosis of this disease, including thoracic surgery, radical radiotherapy, microwave ablation, systemic therapies and cancer immunotherapy^6,7^. Most lung cancer patients present with advanced (stage III or IV) disease at the time of diagnosis, resulting in a 5-year survival rate of 4–17% for all lung cancer subtypes^4^. Thus, there is an urgent need to explore novel early diagnostic markers and therapeutic targets for NSCLC.

RAD21 cohesin complex component (RAD21) is a component of the cohesin complex that regulates sister chromosome separation during late mitosis. RAD21 has also been implicated in the occurrence and development of various cancers^8,9^. For example, RAD21 has been reported to regulate p53 and exportin 1 (XPO1) expression in cervical cancer cells, and its elevated expression was positively correlated with poorer prognosis of patients with this cancer^10^. Similarly, another study using high-throughput sequencing demonstrated a strong correlation between high levels of RAD21 in bladder cancer tissues and poor prognosis of bladder cancer patients^11^. Moreover, a recent study suggested that higher RAD21 expression in NSCLC tissues was associated with poor overall survival of patients with this disease^12^. However, the role of RAD21 in NSCLC is not completely understood.

Nipped-B-like protein (NIPBL) is a cohesin loading factor that binds with human cohesin and DNA to regulate chromosome condensation, genome stability and DNA repair^13,14^. NIPBL also facilitates enhancer-promoter interactions to regulate genome compartmentalization^15^ and has been reported to play a critical role in lung cancer. For instance, inhibition of NIPBL prevented chemotherapeutic resistance by inducing DNA damage in NSCLC^16,17^. Moreover, higher expression of NIPBL in cancer tissues was strongly associated with poorer prognosis of NSCLC patients^17^. However, the correlation between NIPBL and RAD21 in NSCLC has yet to be elucidated.

The phosphoinositide 3-kinase (PI3K) pathway is considered a master regulator of various cancers, including lung cancer^18,19^. As inhibition of this pathway results in the repression of tumor progression^18,20^, some PI3K inhibitors have been approved for cancer treatment. Idelalisib (Zydelig), Copanlisib (Aliqopa) and Duvelisib (Copiktra) have been approved for refractory chronic lymphocytic leukemia (CLL) or relapsed follicular lymphoma (FL) treatment^20^. Moreover, the efficacy of PI3K inhibitors such as CUDC-907 and LY3023414 is being tested in ongoing clinical trials for NSCLC^18,20^.

However, the mechanistic effect of RAD21 or NIPBL in cancers, including NSCLC, is largely unclear, and further studies are needed to determine whether RAD21 or NIPBL could be an indicator for the use of PI3K inhibitors in NSCLC. Therefore, the primary aim of this study was to further investigate whether RAD21 exerts effects through the PI3K pathway and reveal the relationship between RAD21 and NIPBL in NSCLC.

## Methods

### Data collection and analyses

Gene expression profiles and survival information of NSCLC patients were obtained from the Oncomine and The Cancer Genome Atlas (TCGA) databases. Kaplan‒Meier survival curves and the log-rank test were performed to determine the differences in survival rates between NSCLC patients with low and high *RAD21* expression.

### Tissue collection

NSCLC tissues and paracarcinoma tissues were collected from 64 patients treated at the Zhejiang Cancer Hospital from 2008 to 2009. Clinical data of these patients, including sex, age, family history of cancer, and survival time, were also collected. Routine follow-up examinations were conducted every 3 months. All experimental procedures in humans were approved by the Ethics Committee of Zhejiang Cancer Hospital. Written informed consent was obtained from all participants enrolled in this study. Subsequently, the correlation between *RAD21* expression and clinicopathological features or survival of these NSCLC patients was analyzed based on the data collected from the Oncomine and TCGA databases. All ethical regulations relevant to human research participants were followed.

### Quantitative reverse transcription-PCR (qRT‒PCR)

Total RNA from human NSCLC tumor tissues or cells was extracted using TRIzol (Invitrogen, Carlsbad, CA, USA). First-strand cDNA was made according to the manufacturer’s instructions (Takara Biotechnology, China). The results of the target RNA were normalized to that of the internal control (GAPDH) and presented as 2^−▵▵Ct^ values relative to the control sample. qRT‒PCR was performed by SYBR Green (Takara Biotechnology, China) using the following primer sequences: RAD21 forward: 5′-CCACTTGATGGGTTGTCTCTT-3′, reverse: 5′-CGTTAGGGAAATGCTCCAGA-3′; GAPDH forward: 5′-AACGGATTTGGTCGTATTGGG-3′, reverse: 5′-CCTGGAAGATGGTGATGGGAT-3′; NIPBL forward: 5′- CCTCCAGACCTACCTACAAGAA-3′, reverse: 5′- CTCATCCCTGAGGAAACATCAC-3′; GAPDH forward: 5′-AACGGATTTGGTCGTATTGGG-3′, reverse: 5′-CCTGGAAGATGGTGATGGGAT-3′.

### Immunohistochemistry (IHC)

IHC for RAD21 (#ab217678, diluted at 1:300, Abcam, Cambridge, UK) was performed on surgically resected NSCLC tumor and paracarcinoma tissues collected from the Zhejiang cohort with detailed follow-up information as reported earlier^21^.

### Cell culture

The normal human lung fibroblast cell line MRC-5 and human NSCLC cell Lines H1299, H1650, H661, SW900, SK-MES-1, H1703, LK2 and A549 were obtained from the Cell Bank at the Chinese Academy of Sciences (Shanghai, China). All cells were cultured in RPMI-1640 medium (HyClone, Logan, UT, USA) supplemented with 10% FBS, 100 U/mL penicillin and 100 μg/mL streptomycin at 37 °C in a humidified atmosphere of 5% CO_2_.

### Construction of stable cell lines

In this study, a short hairpin RNA (shRNA) lentiviral vector was utilized to establish stably silenced RAD21 lung cancer cell lines. Briefly, control shRNA lentiviral particles were purchased from Santa Cruz (sc-108080). RAD21 shRNA lentiviral particles were constructed by Abm lnc (Zhenjiang, China). Scramble and shRNA lentiviral particles were packaged in lung cancer cell lines. Recipient cell lines were exposed to conditioned medium containing viruses supplemented with 5 μg/mL polybrene for 48 h. Transfected cells were selected with puromycin to generate stable cell lines with RAD21 knockdown (RAD21-KD). RAD21-KD in the stable cell lines was verified by performing qRT‒PCR and Western blotting.

### Cell transfection

Small interfering RNAs (siRNAs) synthesized by Sangon (Shanghai, China) or vectors were transfected into H1299 and H1650 cells by a Lipofectamine 2000 Kit (Thermo Fisher Scientific, Waltham, MA, USA) according to the manufacturer’s protocol. At 48 h post transfection, the cells were collected and used for subsequent experiments as specified. Sequences of siRNAs were listed in Supplementary Table 1.

### Cell Counting Kit-8 (CCK-8) assay

A CCK-8 assay was performed to detect the proliferation of H1299 and H1650 cells. Both cell lines were seeded in 96-well plates at a density of 1 × 10^4^ cells per well. Ten microliters of CCK-8 solution (Beyotime Biotechnology, Shanghai, China) at a 10× dilution was added to each well and incubated at 37 °C for 2 h. Subsequently, absorbance at 450 nm was detected by a Multiskan MK3 microplate reader (Thermo Fisher Scientific). The rate of cell proliferation was calculated according to the following formula: cell proliferation rate (%) = [optical density (OD) of treatment group/OD of control group] ×100%.

### Flow cytometric analysis for cell apoptosis

H1299 and H1650 cells were collected and washed twice with PBS before being resuspended in 100 μL of incubation buffer (10 mM HEPES/NaOH, pH 7.4, 140 mmol/L NaCl, 5 mM CaCl_2_) containing 10 μL Annexin V-FITC and moderate propidium iodide (PI) (Thermo Fisher Scientific). After incubation at room temperature (RT, 25 °C) for 15 min, the cells were analyzed by flow cytometry (FACSARIA, BD Biosciences, San Jose, CA, USA) as previously described^22,23^.

### Transwell assay for cell migration and invasion

Transwell chambers with 8 μm pore polycarbonate membrane inserts (BD Biosciences) were used for this assay. Fifty microliters of Matrigel was coated on the inner side of the inserts, while fibronectin was coated on the other side. After starvation by incubation with serum-free medium for 12–16 h, H1299 and H1650 cells were resuspended and added to the upper chambers, while 0.5 mL/well cell growth medium was added to the lower chambers. After 48 h, the transwell chambers were fixed with 4% paraformaldehyde (PFA) at RT for 30 min, and the Matrigel was wiped off with a cotton swab. Crystal violet was added to the Transwell chambers to dye cells at RT for 30 min. Finally, the number of crystal violet-dyed cells in the lower chamber was manually counted under a light microscope.

### Chromatin immunoprecipitation (ChIP)

In brief, H1299 and H1650 cells were crosslinked by 1% formaldehyde for 15 min at RT before the reaction was stopped by adding 1.375 M glycine. Next, the cells were suspended in lysis buffer and sonicated to shear the DNA. The insoluble material was removed by centrifugation. A 25 mg DNA chromatin sample was adjusted to a total volume of 500 mL in 450 mL of dilution buffer containing protease inhibitors. The chromatin samples were then incubated with 1 μg of NIPBL antibody (#ab245539, Abcam), RAD21 antibody (#4321, Cell Signaling Technology, Danvers, MA, USA), H3K27me3 antibody (#21800-1-AP, Proteintech, Rosemont, IL, USA), enhancer of zeste 2; catalytic subunit of polycomb repressive complex 2 (EZH2) antibody (#21800-1-AP, Proteintech) or anti-rabbit IgG antibody (Cell Signaling Technologies) and incubated with protein A/G magnetic beads overnight at 4 °C with gentle rotation. Magnetic beads were collected by a magnetic separation device (Thermo Fisher Scientific) and cleaned using washing buffer. Subsequently, immunoprecipitated DNAs were eluted with 100 μL elution buffer containing Proteinase K at 62 °C for 2 h. The DNAs were then purified using the spin columns and dissolved in the elution buffer. Finally, chromatin DNA was analyzed by PCR or qPCR.

### Electrophoretic mobility shift assay (EMSA)

First, the NIPBL (#ab131913, Abcam) or RAD21 protein (#ab152647, Abcam) was incubated with a NIPBL antibody (#ab245539, Abcam) or RAD21 antibody (#4321, Cell Signaling Technology) for 30 min at RT, followed by incubation with 2 ng biotin-labeled *RAD21* or *PI3K* gene promoter fragment was incubated with in binding buffer for 20 min at RT as previously described^24^. Competition experiments were performed using linear pUC19. Then mixes were loaded directly onto 5% polyacrylamide pre-run gel in 40 mM Tris–acetate, 2.5 mM EDTA (pH 7.8) and run at 10 V/cm followed by the transfer onto a nylon membrane. Next, the membrane was incubated with streptavidin-conjugated horseradish peroxidase and subsequently with reagents of the ECL Plus Reagent (#PD004-2, Research-bio, Shanghai, China). Finally, the membrane was then developed by X-ray.

### Dual-luciferase reporter gene assay

Different regions of the *RAD21* gene promoter were inserted into luciferase reporter gene vectors. After cotransfection with luciferase vectors and siRNA negative control (NC) vector or NIPBL siRNA, H1299 and H1650 cells were seeded into 24-well plates, and luciferase activity was determined using the Dual Luciferase Reporter Assay System (Promega, Madison, WI, USA) at 48 h after transfection.

### Coimmunoprecipitation (Co-IP)

H1299 and H1650 cells were lysed in nondenaturing lysis buffer. Next, the supernatant of the cell lysate was precleaned by protein A/G magnetic beads (Thermo Fisher Scientific) for 2 h at 4 °C. Subsequently, approximately 300 μg of protein sample was incubated overnight at 4 °C with 1 μg of anti-NIPBL antibody (#ab245539, Abcam) or anti-RAD21 antibody (#4321, Cell Signaling Technology) and 25 μL of protein A/G magnetic beads. The next day, the protein A/G magnetic beads were collected using a magnetic separation device (Thermo Fisher Scientific), and the precipitated complexes were cleansed with washing buffer (Thermo Fisher Scientific). Finally, the bound proteins were analyzed by Western blotting using KDM6B antibody (#ab38113, Abcam) or EZH2 antibody (#21800-1-AP, Proteintech). Rabbit IgG was used as the negative control.

For competitive Co-IP, different dosages of NIPBL expression vector (0.5 μg, 1 μg, and 2 μg) was transfected into control H1299 or H1650 cells and then the interaction RAD21 and NIPBL or EZH2 was evaluated by Co-IP using anti-RAD21 antibody (#4321, Cell Signaling Technology) as above described.

### Western blotting

Total proteins were extracted from cells using RIPA buffer (Cell Signaling Technology) and quantified using the BCA Protein Quantification Kit (Abbkine, Wuhan, Hubei, USA). The same amount of protein was loaded and separated by SDS-polyacrylamide gel electrophoresis (SDS‒PAGE) and then transferred to a PVDF membrane. Subsequently, membranes were blocked with 5% nonfat milk for 1 h at RT and then incubated overnight at 4 °C with the primary antibody. The membranes were then washed with Tris-buffered saline containing 0.1% Tween 20 (TBST) and incubated with the corresponding secondary antibodies (1: 5000, BOSTER, Wuhan, Hubei, China) at RT for 1 h. Finally, the signals of the targeted proteins were detected by a chemiluminescence detection kit (Beyotime Biotechnology). The primary antibodies used in this study included NIPBL antibody (#ab245539, Abcam), RAD21 antibody (#4321, Cell Signaling Technology), lysine demethylase 6B (KDM6B) antibody (#ab38113, Abcam), EZH2 antibody (#21800-1-AP, Proteintech), PI3K antibody (#bs-2067R, Bioss, Beijing, China), phospho-PI3K (Tyr317) antibody (#bs-5570R, Bioss) and GAPDH (#KC-5G5, Aksomicks, Shanghai, China).

### RNA sequencing

After the extraction of total RNA from H1299 and H1650 cells by TRIzol (Invitrogen), cDNA libraries were constructed and sequenced using Illumina NextSeq 500. Clean reads (clean data) with high quality were obtained, and the levels of mRNAs were calculated to identify differentially expressed genes (DEGs) in *RAD21*-silenced cells compared to control cells. Subsequently, DEGs were mapped to GO terms based on the Gene Ontology (GO) database, and gene numbers were calculated for every GO term. KEGG is the major public pathway-related database. Thus, KEGG was used for pathway enrichment analysis to identify enriched metabolic pathways or signal transduction pathways in DEGs.

### Methylated RNA Immunoprecipitation (MeRIP)

H1299 and H1650 cells were collected and lysed, and the nucleic acid fragments were interrupted by ultrasound. Subsequently, the cell lysates were incubated with m6A antibody (1:500, #ab186773, Abcam) at 4 °C overnight. Next, m6A antibody and methylated RNA fragments were captured by avidin magnetic beads, and the level of methylated RNA was identified by qRT‒PCR.

### RNA decay assays

5 μg/mL actinomycin D (#A9415, Sigma‒Aldrich, St. Louis, MO, USA) was added to treat cells to inhibit DNA transcription, and then the cells were collected at 0, 1, 2, 3, 4 and 5 h after actinomycin D treatment, and the RNA levels were determined by qRT‒PCR. The level of RNA at 0 h after actinomycin D treatment was set as 1, and the level of RNA at other times was calculated as the fold change from the level detected at 0 h. Taking the time after adding actinomycin D as the abscissa and the level of RNA as the ordinate, the slope of the decline curve was calculated by linear regression. Subsequently, the half-life of RNA in each group was calculated according to the relationship of half-life (0.693/slope, when the slope was negative, the formula was -0.693/slope). The longer the half-life, the higher the stability of RNA.

### In vivo assays

Animal protocols were approved by the Institutional Animal Care and Use Committee of Zhejiang Cancer Hospital. Four-week-old male BALB/c nude mice were raised in a pathogen-free environment at the experimental animal center. Xenograft tumor models were established by subcutaneous injection of control and RAD21-KD H1299 or H1650 cells with lentiviral vectors for NIPBL knockdown or RAD21 overexpression into the right dorsal flanks of nude mice. YS-49 (5 mg/kg) was administered to the nude mice by tail vein injection once every three days post subcutaneous injection. The size of the xenograft tumors was measured every three days. The mice were sacrificed at Day 21 post subcutaneous injections, and the weights of the xenograft tumors were harvested and measured. We have complied with all relevant ethical regulations for animal use.

### Statistics and reproducibility

Statistical analyses were performed using SPSS (version 21.0, IBM Corp., Armonk, NY, USA). All statistical tests were two-sided, and *P* values of <0.05 were considered statistically significant. Comparison of continuous variables between two groups was carried out using the Mann‒Whitney *U* test and Student’s *t* tests. Statistical differences among three or more groups were examined by the Kruskal‒Wallis test. Kaplan‒Meier survival curves and the log-rank test were performed to determine the differences in the survival rates between the two groups. A multivariate logistic regression analysis was performed based on the patients’ clinicopathological features and RAD21 expression in lung cancer patients.

### Reporting summary

Further information on research design is available in the Nature Portfolio Reporting Summary linked to this article.

## Results

### RAD21 is upregulated in NSCLC patient tissues and cell lines

**Fig. 1:**
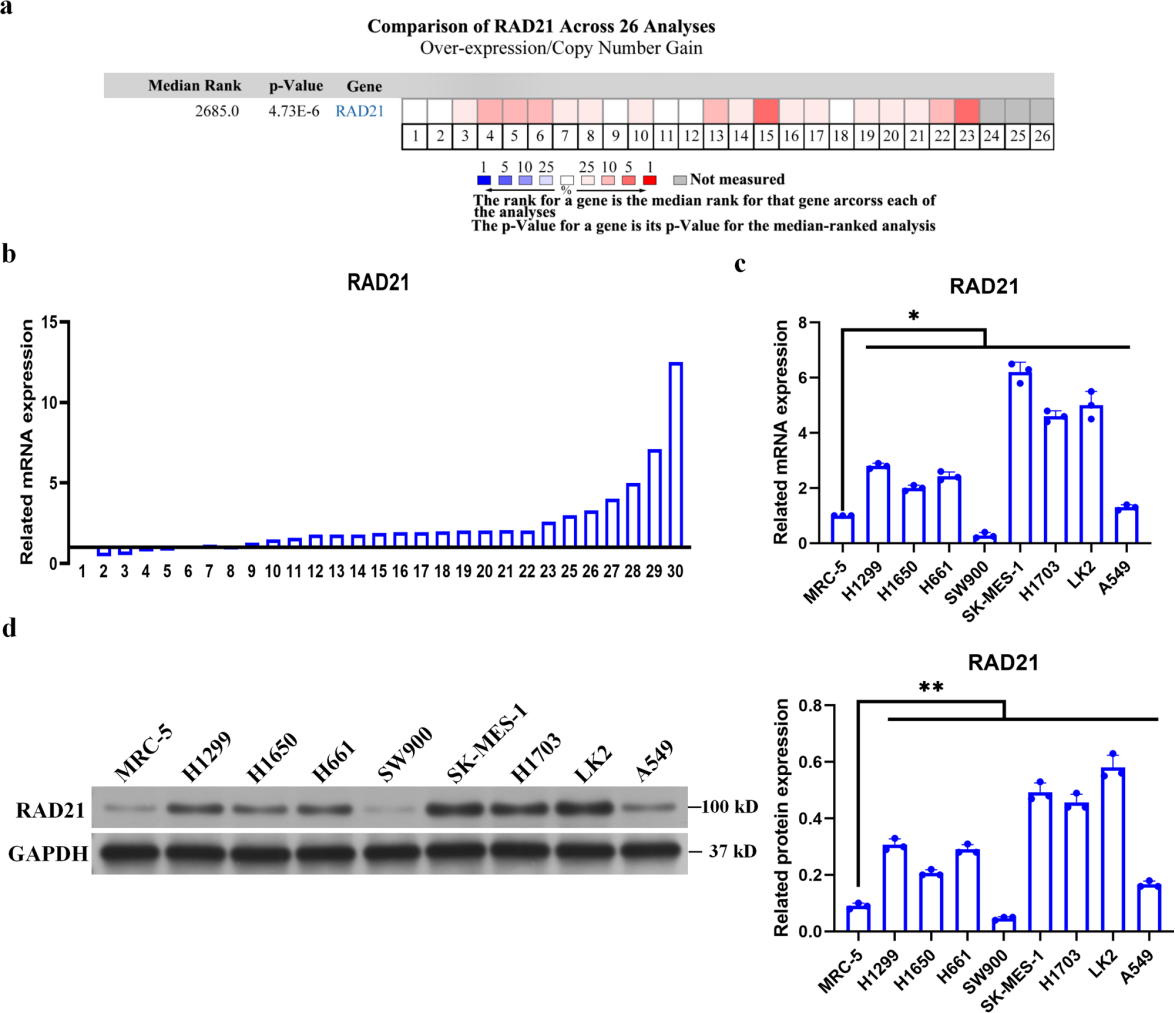
RAD21 is upregulated in NSCLC tissues and cell lines. **a** Data of RAD21 mRNA level in NSCLC tissues obtained from 26 gene expression profiles of NSCLC in Oncomine database. **b** RAD21 mRNA levels detected in NSCLC tissues and paracarcinoma tissues collected from 30 patients treated at Zhejiang Cancer Hospital. **c** RAD21 mRNA levels detected in lung cancer cell lines versus that detected in control MRC-5 cells. **d** RAD21 protein levels detected in lung cancer cell lines versus that detected in control MRC-5 cells. Error bars indicate the standard error of the mean (SEM). *N* = 3. *P < 0.05, **P < 0.01.

Of the 26 gene expression profiles of NSCLC available in the Oncomine database, 17 were confirmed to demonstrate higher RAD21 mRNA levels in NSCLC tissues than in paracarcinoma tissues (*P* < 0.01) (Fig. 1a). To further confirm these results, RAD21 mRNA levels were determined in NSCLC tissues and paracarcinoma tissues collected from 30 patients in the Zhejiang cohort by qRT‒PCR. The results showed that the level of RAD21 mRNA in the tumor tissues from 23 NSCLC patients was higher than that in paracarcinoma tissues (Fig. 1b). Moreover, RAD21 mRNA expression was upregulated in most NSCLC cell lines (including H1299, H1650, H661, SK-MES-1, H1703, LK2 and A549 cells) compared with MRC-5 cells (Fig. 1c). In addition, RAD21 protein expression was increased in H1299, H1650, H661, SK-MES-1, H1703, LK2 and A549 cells compared with MRC-5 cells (Fig. 1d). Therefore, these results suggest that RAD21 is upregulated in NSCLC tissues and cell lines.

### Correlation between OS or clinicopathological features and RAD21 expression in NSCLC patients

**Fig. 2:**
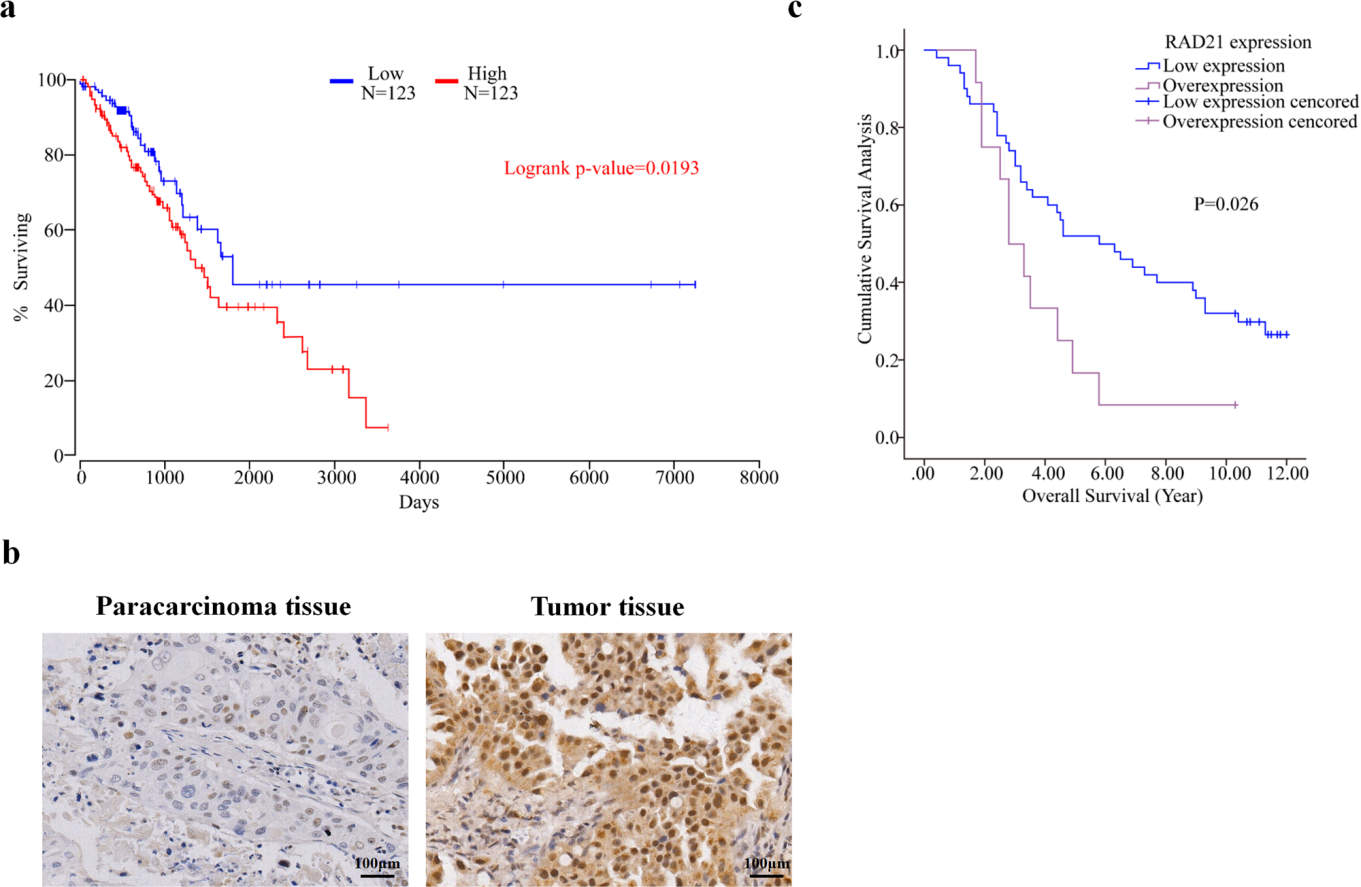
Correlation between OS or clinicopathological features and RAD21 expression in NSCLC patients. **a** Kaplan–Meier survival curves demonstrating the correlation between RAD21 expression and OS of NSCLC patients collected from the TCGA database. *N* = 246. **b** Representative IHC images of RAD21 protein expression in NSCLC tissues and paracarcinoma tissues collected from 64 patients treated at the Zhejiang Cancer Hospital. **c** Kaplan-meier survival curves demonstrating the correlation between RAD21 expression and OS of NSCLC patients from the Zhejiang cohort. Bar = 100 μm. *N* = 64.

The differences in the survival rates between patients with low and high RAD21 mRNA expression were determined using data sourced from 246 NSCLC patients in the TCGA database. The results showed that the overall survival (OS) of NSCLC patients with high RAD21 mRNA expression was dramatically shorter than that of NSCLC patients with low RAD21 mRNA expression (*P* = 0.0193) (Fig. 2a).

To further confirm the correlation between RAD21 expression and the OS of NSCLC patients, RAD21 protein expression was detected by IHC in NSCLC tissues and paracarcinoma tissues collected from the Zhejiang cohort (Fig. 2b). Consistent with the TCGA results, our IHC results also showed a positive correlation between higher RAD21 expression and shorter OS of NSCLC patients when compared to their counterparts with lower RAD21 protein expression (*P* = 0.026) (Fig. 2c). Together, these results suggest that RAD21 is closely related to the poorer OS of patients with NSCLC, which was consistent with findings reported in a previous study^12^.

To further determine the prognostic value of RAD21 expression in NSCLC patients, multivariate analyses were performed, and the results revealed that RAD21 expression was an independent prognostic factor for OS in NSCLC patients (Supplementary Table 2). Although the correlation between clinicopathological features and RAD21 expression was also analyzed for the Zhejiang cohort, no associations could be determined between RAD21 expression and the clinicopathological features of these NSCLC patients (Supplementary Table 3).

### Effects of RAD21 on NSCLC cells in vitro

**Fig. 3:**
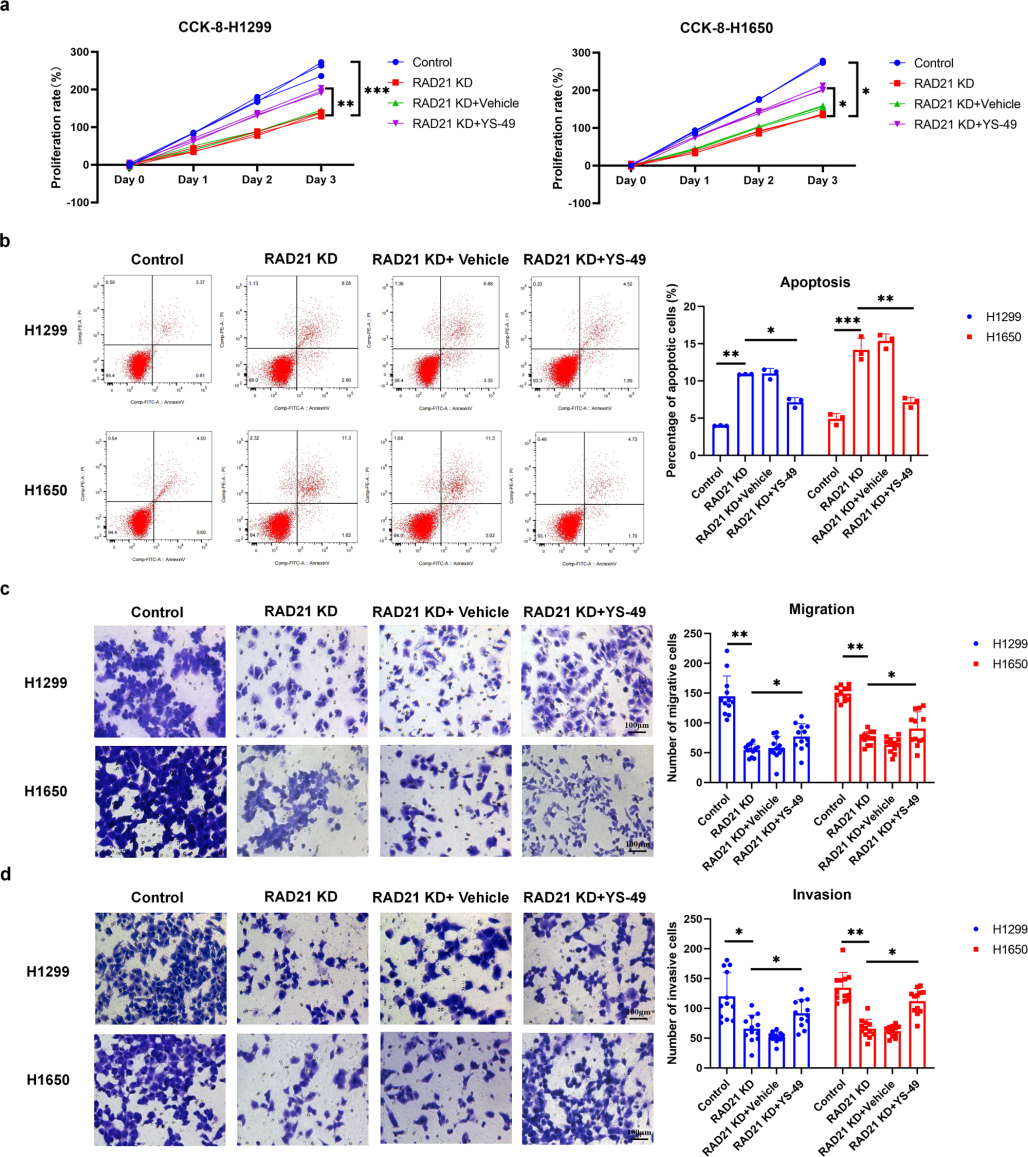
RAD21 exerts effects through the PI3K pathway in NSCLC cells. **a** Proliferation rates of control vector and RAD21-KD H1299 and H1650 transfected cells treated with or without YS-49. *N* = 3. **b** Representative images of flow cytometric apoptosis assay in RAD21 knockdown H1299 and H1650 cells treated with or without YS-49. *N* = 3. **c** Representative images and quantification of migratory RAD21-KD H1299 and H1650 cells treated with or without YS-49. *N* = 3. **d** Representative images and quantification of invasive RAD21-KD H1299 and H1650 cells treated with or without YS-49. Bar = 100μm. Error bars indicate the standard error of the mean (SEM). *N* = 3. KD: knockdown. *P < 0.05, **P < 0.01, ***P < 0.001.

To date, the effects of RAD21 on NSCLC cells remain unclear. Therefore, we generated RAD21 knockdown using shRNA in H1299 and H1650 cells. Following confirmation of the reduction in RAD21 mRNA levels in the RAD21-KD cells (Supplementary Fig. 1), CCK-8 assays were performed to determine the effect of RAD21-KD on the proliferative abilities of the NSCLC cell lines. The results showed that knockdown of RAD21 dramatically suppressed the proliferation of both cell lines (Fig. 3a). Consistently, flow cytometric analysis showed that RAD21-KD significantly induced apoptosis in both H1299 and H1650 cells (Fig. 3b). Moreover, a transwell assay demonstrated that knockdown of RAD21 reduced the number of migratory and invasive cells (Fig. 3c, d). Overall, these results suggest that RAD21 promotes NSCLC cell proliferation, migration and invasion by preventing NSCLC cell apoptosis.

### RAD21 enhances *PI3K* gene transcription as a transcription factor in NSCLC cells

**Fig. 4:**
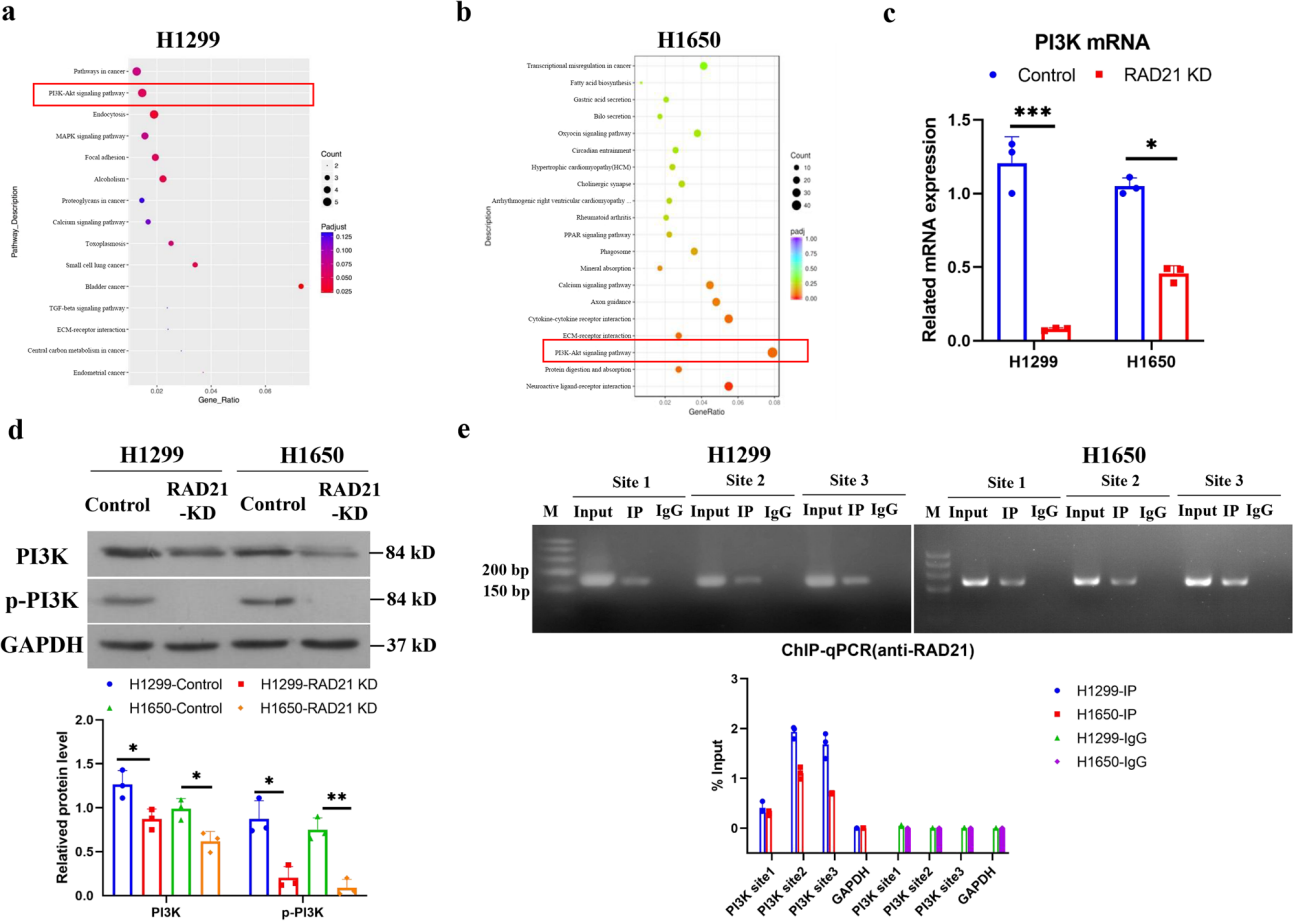
RAD21 enhances *PI3K* gene transcription as a transcription factor in NSCLC cells. Top enriched KEGG pathways of DEGs in RAD21-silenced stably transfected H1299 cells (**a**) and H1650 cells (**b**). **c** PI3K mRNA levels in control and RAD21-silenced stably transfected H1299 and H1650 cells. *N* = 3. **d** PI3K and p-PI3K protein levels in control and RAD21-silenced stably transfected H1299 and H1650 cells. *N* = 3. **e** Immunoprecipitated chromatin was analyzed by PCR and qPCR for the *PI3K* gene promoter in H1299 and H1650 cells. KD knockdown. Error bars indicate the standard error of the mean (SEM). *P < 0.05, **P < 0.01, ***P < 0.001.

To further explore downstream target genes or pathways of RAD21 in NSCLC, RNA sequencing in RAD21-KD H1299 and H1650 cells was performed. Pathway analysis of differentially expressed genes (DEGs) demonstrated enrichment of the PI3K-AKT serine/threonine kinase (AKT) pathway in both RAD21-KD H1299 and H1650 cells (Fig. 4a, b). As the PI3K-AKT pathway is known to regulate cell proliferation, cell death and motility to facilitate cancer development in various cancers^19,20,25^, our results indicate that PI3K might be a potential downstream target of RAD21 in NSCLC.

Subsequent analyses revealed decreased PI3K mRNA and protein levels in RAD21-KD cells compared to control vector-transfected cells (Fig. 4c, d)^8^. As RAD21 binds to chromosome together with CTCF^26,27^, RAD21/CTCF binding sites on *PI3K* gene promoter were explored. Prediction by the JASPAR website (https://jaspar.genereg.net/) showed the potential RAD21/CTCF binding sites on the human *PI3K* gene promoter (Supplementary Figure 2). Furthermore, ChIP performed by RAD21 antibody confirmed that RAD21 protein bound to the *PI3K* gene promoter in H1299 and H1650 cells (Fig. 4e). In addition, EMSA using a probe corresponding to *PI3K* gene promoter together with RAD21 protein detected a DNA-protein complex band, while adding the RAD21 antibody led to a super-shift of DNA-protein complex band (Supplementary Fig. 3a), indicating the DNA binding ability of RAD21 with *PI3K* gene promoter in vitro. Together, these results suggest that RAD21 promotes *PI3K* gene transcription directly by acting on its gene promoter in NSCLC cells.

### RAD21 exerts effects through the PI3K pathway in NSCLC cells

RAD21 facilitates *PI3K* gene transcription, and the PI3K pathway is an important regulator in lung cancer tumorigenesis^18,19^. We next sought to test whether RAD21 exerts its effects on NSCLC cells through the PI3K pathway. Western blot results showed that RAD21 protein levels were decreased in RAD21-KD H1299 and H1650 cells (Supplementary Fig. 4). Knockdown of RAD21 also reduced phosphorylated PI3K and total protein levels of other factors involved in the PI3K pathway, including AKT, Extracellular signal-regulated kinase 1 (ERK1) and mitogen-activated protein kinase (MAPK) (Supplementary Fig. 4). These data suggest that RAD21 regulates the activity of the PI3K pathway in NSCLC cells.

Next, the PI3K activator YS-49 was used to further confirm whether RAD21 exerts effects on NSCLC cells by the PI3K pathway. The results of the CCK-8 assay demonstrated that YS-49 reversed the inhibitory proliferative effects of RAD21-KD on H1299 and H1650 cells (Fig. 3a). In contrast, flow cytometric analysis indicated that YS-49 prevented RAD21-KD-mediated induction of apoptosis in transfected H1299 and H1650 cells (Fig. 3b). Moreover, a transwell assay demonstrated that YS-49 also neutralized the inhibitory effect of RAD21-KD on the migration and invasion of H1299 and H1650 cells (Fig. 3c, d). Overall, these results suggest that RAD21 mediates its effects via the PI3K pathway in NSCLC cells.

### NIPBL enhances *RAD21* gene transcription in NSCLC cells

**Fig. 5:**
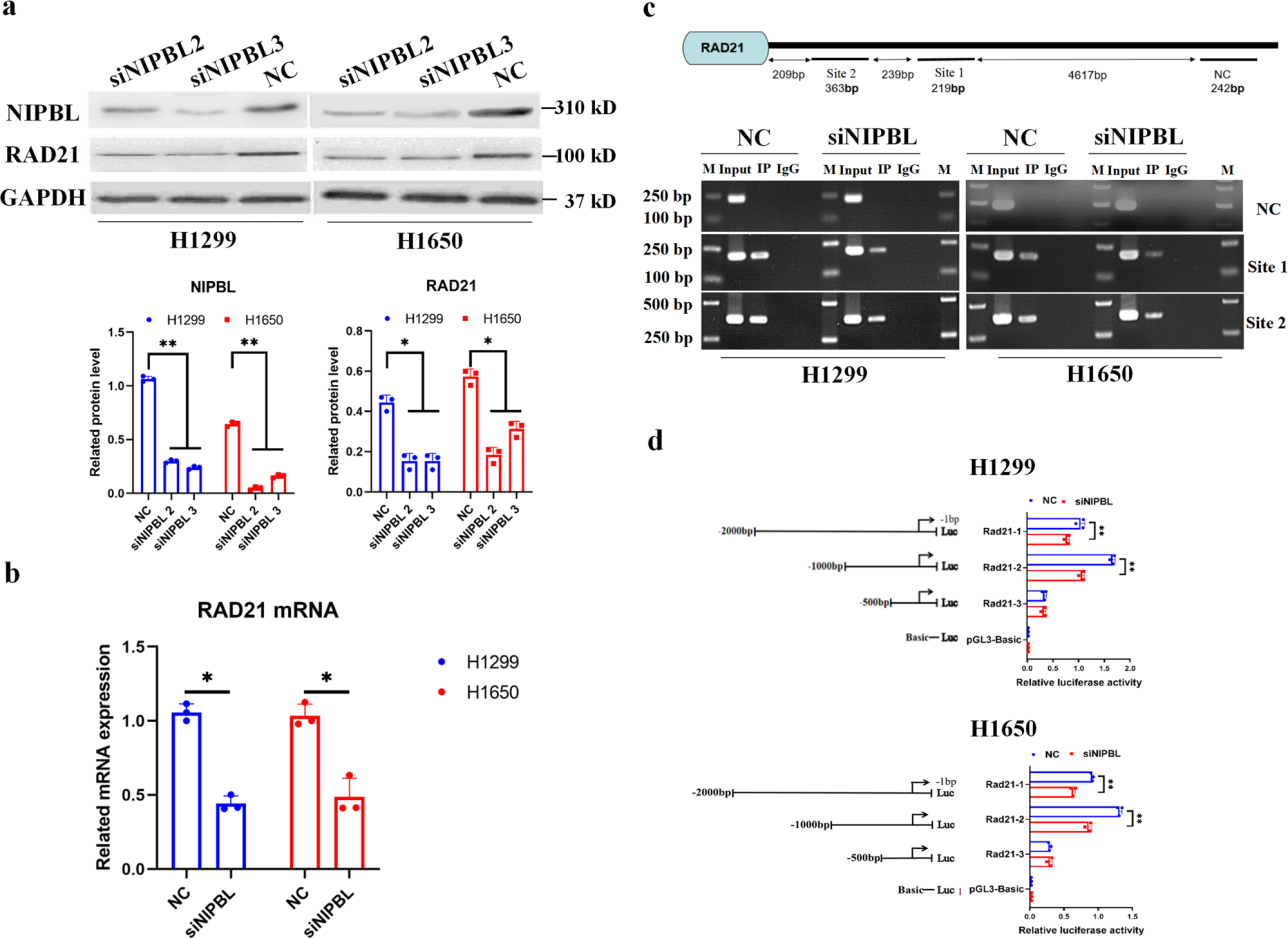
NIPBL enhances *RAD21* gene transcription in NSCLC cells. **a** NIPBL protein expression and (**b**) RAD21 mRNA levels in scrambled and NIPBL siRNA transfected H1299 and H1650 cells. **c** Immunoprecipitated chromatin was analyzed by PCR for the *RAD21* gene promoter in scrambled and NIPBL siRNA transfected H1299 and H1650 cells. **d** Translation activity of *RAD21* gene was analyzed by relative luciferase reporter activity assay in scrambled and NIPBL siRNA transfected H1299 and H1650 cells. NC negative control; siNIPBL, NIPBL siRNA. Error bars indicate the standard error of the mean (SEM). *N* = 3. *P < 0.05, **P < 0.01.

To date, the correlation between NIPBL and RAD21 in NSCLC has yet to be elucidated. To identify the effect of NIPBL on RAD21 expression, siRNA was used to silence NIPBL expression in H1299 and H1650 cells. Transfection of NIPBL siRNAs effectively reduced NIPBL expression in H1299 cells and H1650 cells (Fig. 5a). The results showed that downregulation of NIPBL resulted in a corresponding decrease in RAD21 mRNA and protein levels (Fig. 5a, b). As NIPBL binds with DNA to facilitate enhancer-promoter communication, the interaction between the NIPBL protein and the RAD21 promoter was determined by ChIP using an anti-NIPBL antibody. Besides, EMSA using a probe corresponding to *RAD21* gene promoter together with NIPBL protein determined a DNA-protein complex band, while adding the NIPBL antibody resulted in a super-shift of DNA-protein complex band (Supplementary Fig. 3b), indicating the DNA binding ability of NIPBL with *RAD21* gene promoter in vitro. The results indicated interactions between the NIPBL protein and the RAD21 promoter in both H1299 and H1650 cells (Fig. 5c). Subsequently, a dual-luciferase reporter gene assay revealed that downregulation of NIPBL suppressed *RAD21* gene transcription in H1299 and H1650 cells, and the binding site of NIPBL on the *RAD21* gene promoter was predicted to be located between approximately −2000 bp and −1000 bp (Fig. 5d). These data suggest that NIPBL enhanced RAD21 expression by promoting its transcription.

### NIPBL promotes *RAD21* gene transcription by enhancing H3K27 demethylation by recruiting KDM6B to the *RAD21* gene promoter in NSCLC cells

**Fig. 6:**
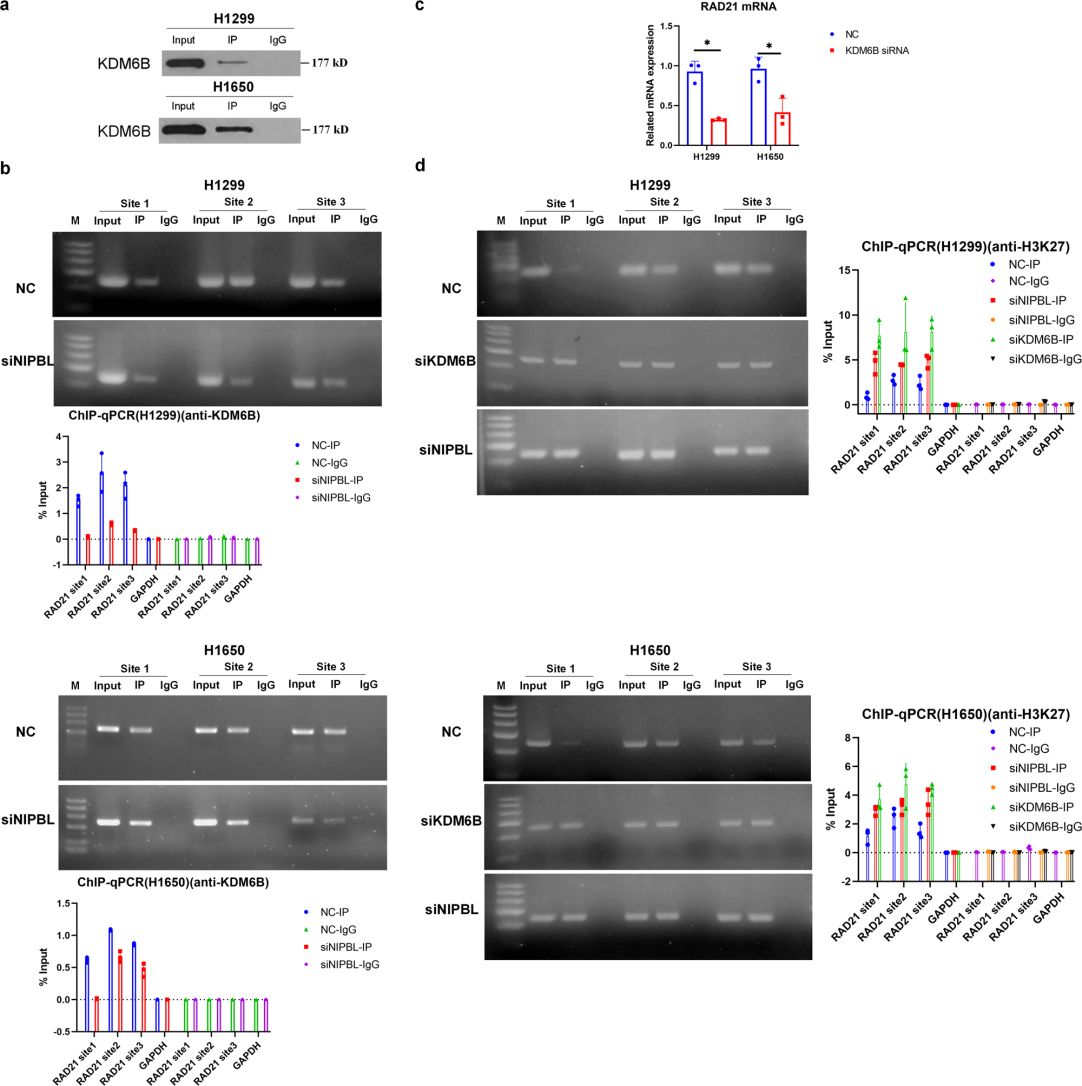
NIPBL promotes *RAD21* gene transcription through enhancing H3K27 demethylation via recruiting KDM6B to *RAD21* gene promoter in NSCLC cells. **a** Representative image of Co-IP using an anti-NIPBL antibody in H1299 and H1650 cells. Rabbit IgG was used as negative control. **b** Immunoprecipitated chromatin was analyzed by PCR and qPCR for the *RAD21* gene promoter in scrambled and NIPBL siRNA transfected H1299 and H1650 cells. **c** RAD21 mRNA expression detected in scrambled and KDM6B siRNA transfected H1299 and H1650 cells. *N* = 3. **d** Immunoprecipitated chromatin was analyzed by PCR and qPCR for the *RAD21* gene promoter in scrambled and NIPBL or KDM6B siRNA transfected H1299 and H1650 cells. NC negative control; siKDM6B, KDM6B siRNA. Error bars indicate the standard error of the mean (SEM). *P < 0.05.

We next explored the mechanism by which NIPBL regulates *RAD21* gene transcription. A previous study suggested that the NIPBL protein interacts with KDM6B in leukemia cells^28^. KDM6B loosens the chromatin structure to promote the expression of target genes by removing H3K27 methylation^29–31^. We performed Co-IPs to determine whether NIPBL similarly interacts with KDM6B in lung cancer cells. The results indicated that NIPBL interacted with KDM6B in both H1299 and H1650 cells (Fig. 6a). As we have previously shown that NIPBL binds to the *RAD21* gene promoter, we performed ChIP assays to determine whether NIPBL mediates the binding of KDM6B to the *RAD21* gene promoter. The results showed that KDM6B bound to the *RAD21* gene promoter, whereas downregulation of NIPBL inhibited this association in H1299 and H1650 cells (Fig. 6b), thereby confirming that NIPBL facilitates the binding of KDM6B to the *RAD21* gene promoter.

Next, we determined the effect of KDM6B knockdown on RAD21 expression. After confirming the downregulation of KDM6B mRNA levels in KDM6B siRNA-transfected H1299 and H1650 cells (Supplementary Fig. 5), we then assessed RAD21 mRNA levels in these cells by qRT‒PCR. The results revealed a concomitant reduction in RAD21 mRNA levels in KDM6B siRNA-transfected H1299 and H1650 cells (Fig. 6c), suggesting that KDM6B might enhance *RAD21* gene transcription. Furthermore, ChIP‒qPCR with an anti-H3K27me3 antibody showed that knockdown of both NIPBL and KDM6B reduced the H3K27me level on the *RAD21* gene promoter (Fig. 6d). Together, these results suggested that NIPBL promotes *RAD21* gene transcription by enhancing H3K27 demethylation through its recruitment of KDM6B to the *RAD21* gene promoter in NSCLC cells.

### NIPBL reverses the effect of EZH2 on RAD21-mediated *PI3K* gene transcription by disrupting the association between EZH2 and RAD21 in NSCLC cells

**Fig. 7:**
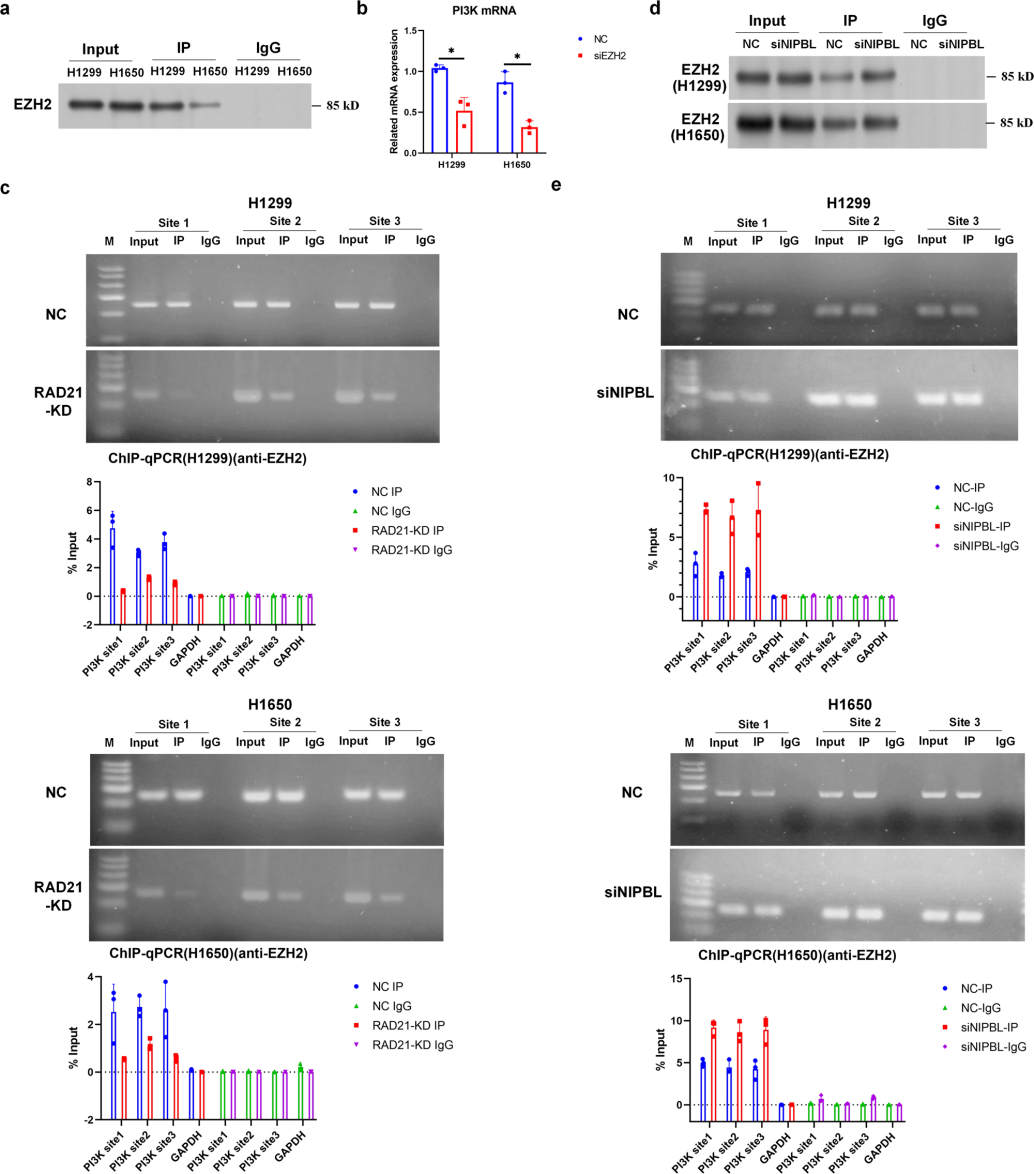
NIPBL reverses the effect of EZH2 on RAD21-mediated *PI3K* gene transcription through disrupting the association between EZH2 and RAD21 in NSCLC cells. **a** Representative image of Co-IP using an anti-RAD21 antibody in H1299 and H1650 cells. Rabbit IgG was used as negative control. *N* = 3. **b** PI3K mRNA expression detected in scrambled and EZH2 siRNA transfected H1299 and H1650 cells. **c** Immunoprecipitated chromatin was analyzed by PCR and qPCR for the *PI3K* gene promoter in H1299 and H1650 cells. **d** Representative images of Co-IP using an anti-RAD21 antibody in scrambled and NIPBL siRNA transfected H1299 and H1650 cells. Rabbit IgG was used as a negative control. **e** Immunoprecipitated chromatin was analyzed by PCR and qPCR for the *PI3K* gene promoter in H1299 and H1650 cells. NC negative control; siEZH2, EZH2 siRNA. Error bars indicate the standard error of the mean (SEM). *P < 0.05.

A previous study demonstrated an association between RAD21 and the histone methylase EZH2 in pluripotent cells^32^. In contrast to KDM6B, EZH2 suppresses the transcription of target genes by enhancing H3K27 methylation^33,34^. Co-IP for RAD21 antibody demonstrated that RAD21 interacted with EZH2 in H1299 and H1650 cells (Fig. 7a). Moreover, downregulation of EZH2 using siRNA (Supplementary Fig. 6) decreased PI3K mRNA levels in H1299 and H1650 cells (Fig. 7b), suggesting that EZH2 might counteract the effect of RAD21 on *PI3K* gene transcription.

Although ChIP performed with an EZH2 antibody indicated that EZH2 binds to the *PI3K* gene promoter, we also observed that silencing RAD21 disrupted this binding (Fig. 7c), indicating that EZH2 binding to the *PI3K* gene promoter is mediated by RAD21. Besides, Co-IP performed by RAD21 antibody showed that downregulation of NIPBL enhanced the interaction of EZH2 and RAD21 (Fig. 7d). Competitive Co-IP performed by RAD21 antibody after transfection of different dosages of NIPBL expression vector further indicated that NIPBL blocked the interaction of RAD21 and EZH2 through competitively binding with RAD21 in H1299 and H1650 cells (Supplementary Fig. 7a, b). In addition, ChIP performed with an EZH2 antibody demonstrated that NIPBL silence promoted the binding of EZH2 to the *PI3K* gene promoter (Fig. 7e). Overall, these results suggested that NIPBL might reverse the effect of EZH2 on RAD21-mediated *PI3K* gene transcription by disrupting the association between EZH2 and RAD21 in NSCLC cells.

### RNA methylation elevates NIPBL mRNA levels by improving NIPBL mRNA stability in NSCLC cells

**Fig. 8:**
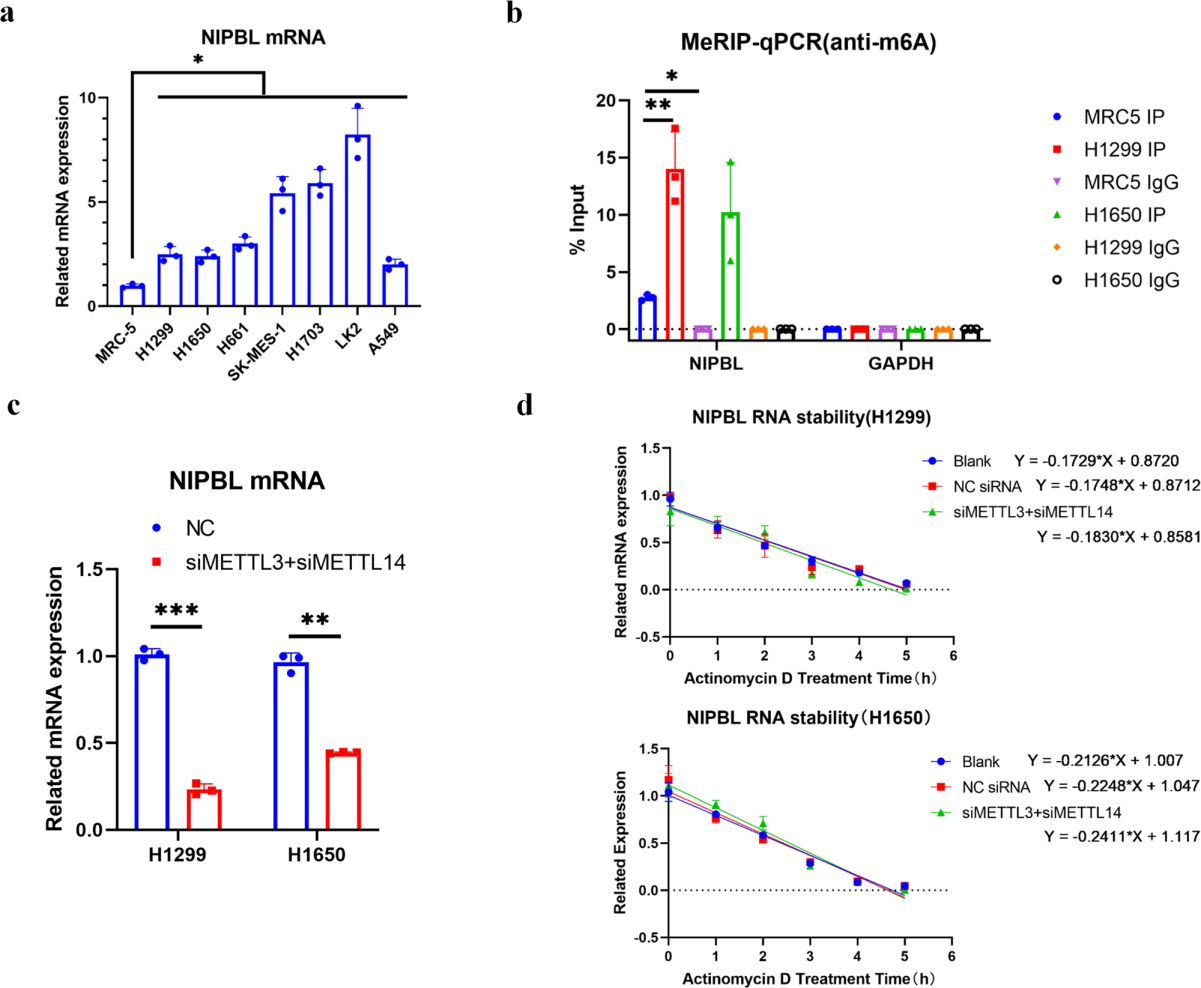
RNA methylation elevates NIPBL mRNA level through improving NIPBL mRNA stability in NSCLC cells. **a** Comparison of NIPBL mRNA levels in lung cancer cell lines versus control MRC-5 cells. *N* = 3. **b** Quantification of methylated NIPBL mRNA by qRT-PCR in MRC-5, H1299 and H1650 cells following MeRIP with m6A antibody. *N* = 3. **c** NIPBL mRNA levels in scrambled, METTL3 and METTL14 siRNA transfected H1299 and H1650 cells. *N* = 3. **d** qPCR results showing NIPBL RNA stability in scrambled, METTL3 and METTL14 siRNA transfected at 0 h, 1 h, 2 h, 3 h, 4 h and 5 h following treatment with actinomycin D. NC negative control; siMETTL3, METTL3 siRNA; siMETTL14, METTL14 siRNA. Error bars indicate the standard error of the mean (SEM). *P < 0.05, **P < 0.01, ***P < 0.001.

Consistent with the RAD21 mRNA level, the NIPBL mRNA level was also upregulated in NSCLC cells (Fig. 8a). RNA methylation is involved in the occurrence and development of various diseases by regulating the stability, localization, transportation, shearing and translation of RNA^35,36^. To explore whether RNA methylation contributed to NIPBL expression, the methylation of NIPBL mRNA was detected by MeRIP. The results demonstrated that the methylation of NIPBL mRNA was dramatically increased in H1299 and H1650 cells compared with MRC-5 cells (Fig. 8b).

To determine the effect of RNA methylation on NIPBL mRNA levels, RNA methylation was suppressed by silencing methyltransferase 3 (METTL3) and methyltransferase 14 (METTL14) using siRNA (Supplementary Fig. 8). Subsequent qRT‒PCR revealed that silencing METTL3 and METTL14 reduced NIPBL mRNA levels (Fig. 8c), suggesting that RNA methylation might increase NIPBL mRNA levels.

As RNA methylation regulates RNA stability, which affects mRNA levels, the half-life of NIPBL mRNA was detected by RNA decay assay in H1299 and H1650 cells. The results demonstrated that the half-life of NIPBL mRNA in the control group of H1299 cells was 3.96 h, which decreased to 3.78 h following silencing of METTL3 and METTL14 (Fig. 8d). Similarly, the half-life of NIPBL mRNA in the control group of H1650 cells was 3.26 h, which was decreased to 2.87 h in METTL3- and METTL14-silenced cells (Fig. 8d). These results suggested that RNA methylation enhances the stability and increases the levels of NIPBL mRNA.

### NIPBL-mediated RAD21 facilitates the tumorigenicity of NSCLC through the PI3K pathway in vivo

**Fig. 9:**
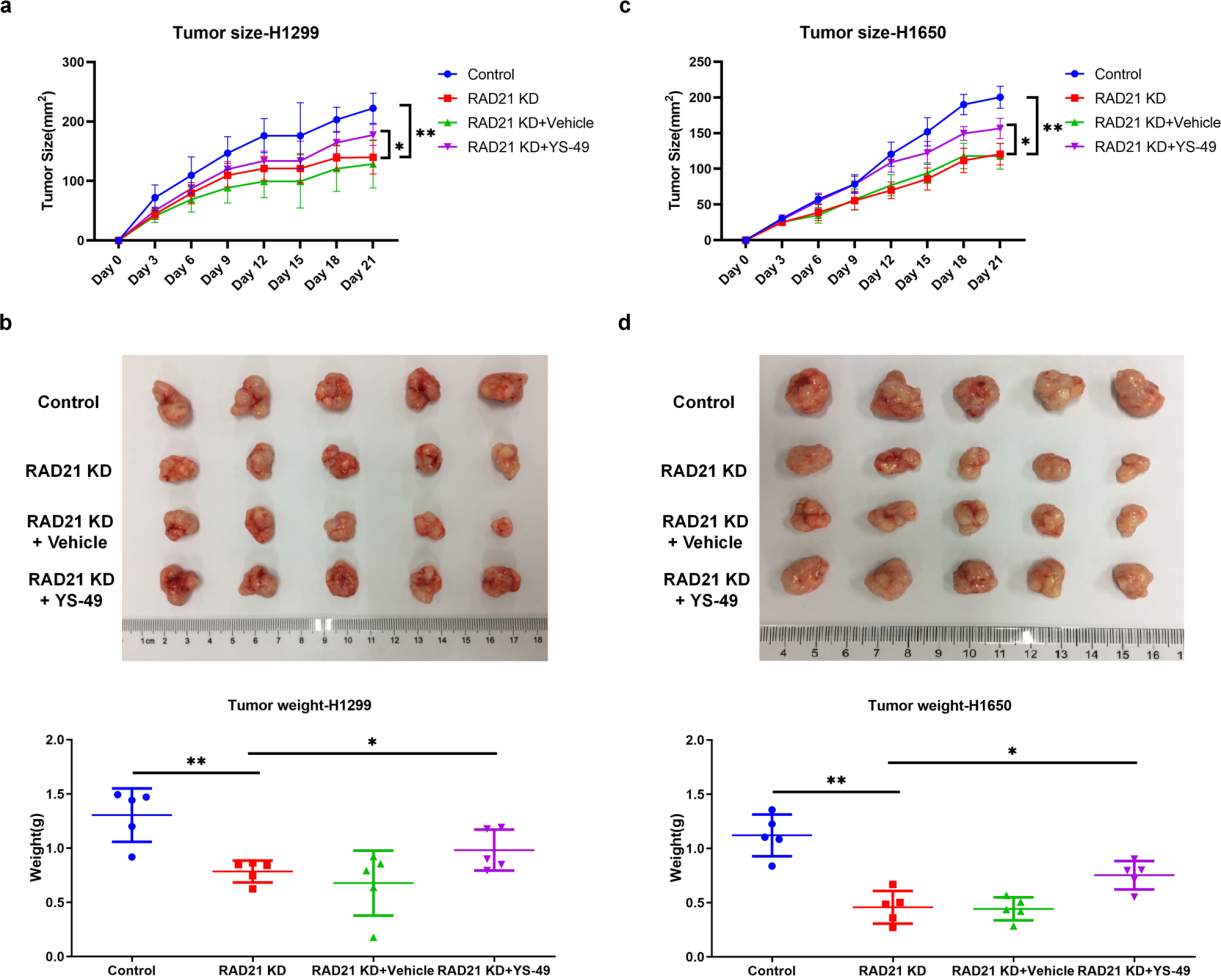
RAD21 facilitates the tumorigenicity of NSCLC through PI3K pathway in vivo. **a**, **c** Quantification of the volume of xenograft tumors derived from control and RAD21-KD H1299 (**a**) and H1650 (**c**) cells in the presence and absence of YS-49. *N* = 5. **b**, **d** Images and quantification of xenograft tumors weight formed in nude mice injected with control and RAD21-KD H1299 (**b**) and H1650 (**d**) cells in the presence and absence of YS-49. KD knockdown. Error bars indicate the standard error of the mean (SEM). *N* = 5. *P < 0.05, **P < 0.01.

To further confirm the oncogenic role of RAD21 in vivo, we established a xenograft tumor mouse model by subcutaneously injecting control and RAD21-KD H1299 or H1650 cells into the right dorsal flanks of nude mice. The results showed that tumors developed from RAD21-KD H1299 (Fig. 9a, b) or H1650 cells (Fig. 9c, d) were smaller and weighed less than tumors from control cells. However, subsequent injection of YS-49 improved the size and weight of RAD21-KD H1299 (Fig. 9a, b) or H1650 (Fig. 9c, d) tumors, suggesting that YS-49 reversed the inhibitory effect of RAD21 knockdown on NSCLC development in vivo.

Besides, NIPBL knockdown (NIPBL-KD) by injection of lentiviral vector dramatically reduced the size and weight of tumors developed from control H1299 (Supplementary Fig. 9a, b) or H1650 cells (Supplementary Fig. 9c, d). However, subsequent injection of lentiviral vector for RAD21 overexpression and YS-49 increased the size and weight of tumors developed from NIPBL-KD H1299 (Supplementary Fig. 9a, b) or H1650 cells (Supplementary Fig. 9c, d), demonstrating that RAD21 overexpression and YS-49 neutralized the inhibitory effect of NIPBL knockdown on NSCLC development in vivo. These results indicate that NIPBL-mediated RAD21 accelerates the in vivo tumorigenicity of NSCLC via the PI3K pathway.

## Discussion

Our results suggest that RAD21 is upregulated in NSCLC tissues and cell lines and that RAD21 expression is an independent prognostic factor for OS in NSCLC patients. Moreover, RAD21 mediates its oncogenic role in NSCLC via activation of the PI3K pathway in vitro and in vivo. Mechanistically, NIPBL promotes *RAD21* gene transcription by enhancing H3K27 demethylation via the recruitment of KDM6B to the *RAD21* gene promoter. Moreover, RAD21 enhances *PI3K* gene transcription by directly binding to its promoter. The interplay between NIPBL and EZH2 in RAD21-mediated PI3K gene transcription is a unique epigenetic mechanism. In addition, RNA methylation elevated NIPBL mRNA levels by improving NIPBL mRNA stability.

RAD21 has been implicated in the occurrence and development of various cancers^8,9^. For example, the high expression of RAD21 has been positively correlated with the poor prognosis of patients with cervical cancer^10^ and bladder cancer^11^. Similarly, elevated RAD21 expression in patients with colorectal cancer has been positively correlated with metastasis and shorter survival^37^. In lung cancer, a recent study indicated that higher levels of RAD21 in NSCLC tissues are associated with poorer survival of patients with NSCLC^12^. However, the precise mechanism by which RAD21 regulates the occurrence and development of cancers remains unclear.

This study revealed the mechanism of RAD21’s protumorigenic roles in NSCLC to the best of our knowledge. Our results suggest that RAD21 promotes lung cancer cell tumorigenicity through activation of the PI3K pathway. The PI3K pathway has been heavily implicated in the tumorigenesis and progression of lung cancer through its regulation of cell proliferation, cell death, migration and invasion^19,38^. For instance, fibroblast growth Factor 21 promotes NSCLC cell growth and migration through PI3K/AKT signaling^39^. Similarly, lncRNA HOXB cluster antisense RNA 3 (HOXB-AS3) activates the PI3K/AKT pathway to enhance cell proliferation, migration, and invasion in lung cancer^40^. Moreover, downstream of tyrosine kinase 7 transcript variant 1 (DOK7V1) contributes to the malignant phenotype of lung cancer cells by regulating the PI3K/AKT pathway^41^. To date, its relationship with RAD21 has not been explored, and this study, to the best of our knowledge, demonstrates that RAD21 mediates its oncogenic effects via the PI3K pathway in lung cancer.

PI3K has been a promising target for cancer treatment^18,20^. To date, several PI3K inhibitors have been employed in clinical trials for NSCLC, such as CUDC-907 and LY3023414^18,20^. However, the PI3K or PI3K pathway is not activated in all NSCLC patients through genetic mutation. Therefore, the identification of biomarkers for the use of PI3K inhibitors in NSCLC patients is needed. Our studies, to the best of our knowledge, indicate an epigenetic mechanism for the activation of PI3K and suggest that RAD21 might be a suitable indicator for the use of PI3K inhibitors in patients with NSCLC.

As a cohesin loading factor, NIPBL binds with human cohesin to compact DNA and regulate chromosome condensation, genome stability and DNA repair^13,14,42^. However, the effect of NIPBL in human cohesin complexes is not entirely clear. Our results reveal that NIPBL promotes *RAD21* gene transcription by enhancing H3K27 demethylation and recruiting KDM6B to the *RAD21* gene promoter. Previous studies have indicated that NIPBL recruits histone deacetylase (HDAC) to trigger histone deacetylation and regulates local chromatin modifications^14,43^. Conversely, histone acetylation facilitates NIPBL occupancy at enhancers to regulate transcription^15^. Therefore, histone modification is essential for NIPBL occupancy at DNA, and subsequently, NIPBL exerts its effect by altering histone modification.

Our results also demonstrated that NIPBL reverses the effect of EZH2 on RAD21-mediated *PI3K* gene transcription by disrupting the association between EZH2 and RAD21 protein. NIPBL usually binds with human cohesin to exert its effects on DNA^13,14^. Therefore, NIPBL might disrupt the association between EZH2 and RAD21 protein through the competitive binding of the RAD21 protein on the *PI3K* gene promoter and subsequently facilitate *PI3K* gene transcription through RAD21.

## Conclusion

**Fig. 10:**
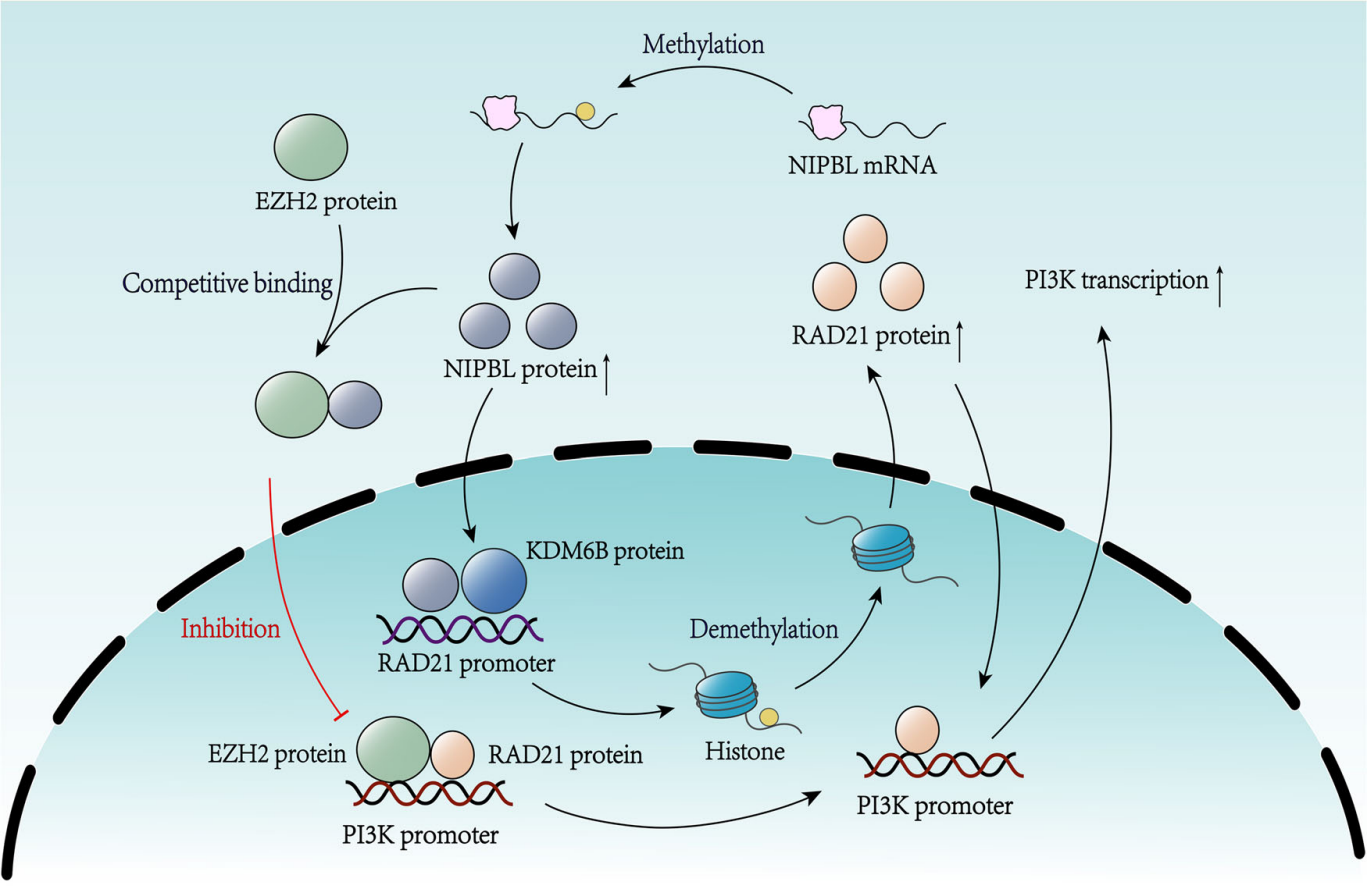
Schematic diagram of molecular mechanisms for the present study. RAD21 mediates its oncogenic role in NSCLC via its activation of the PI3K pathway. Mechanistically, NIPBL promoted *RAD21* gene transcription by enhancing H3K27 demethylation by recruitment of KDM6B to the *RAD21* gene promoter. Moreover, RAD21 enhanced *PI3K* gene transcription by serving as a transcription factor. NIPBL reversed the effect of EZH2 on RAD21-mediated PI3K gene transcription by disrupting the association between EZH2 and RAD21 protein. In addition, RNA methylation elevated NIPBL mRNA level by increasing NIPBL mRNA stability.

In summary, this study demonstrates that RAD21 is upregulated in NSCLC and is an independent prognostic factor for OS in NSCLC patients. RAD21 mediates its tumorigenic effects by activating the PI3K pathway. Mechanistically, NIPBL promotes *RAD21* gene transcription by enhancing H3K27 demethylation and KDM6B recruitment. Concomitantly, NIPBL negates the effect of EZH2 on RAD21-mediated PI3K gene transcription by disrupting the association between EZH2 and the RAD21 protein (Fig. 10). Together, these findings demonstrate the oncogenic role of RAD21 in NSCLC and suggest its use as a potential novel diagnostic marker and therapeutic target for NSCLC.

### Supplementary information

Supplementary Information

Description of Additional Supplementary Files

Supplementary Data 1

Reporting Summary

## Data Availability

Gating strategy is present in Supplementary Figure 10 and 11. Uncropped and unedited blot/gel images have been present in Supplementary Information. The datasets used and analyzed during the current study are available from Supplementary Data 1. RNA sequencing data are available at SRA (PRJNA1062116).
