# Author Correction: NIPBL-mediated RAD21 facilitates tumorigenicity by the PI3K pathway in non-small-cell lung cancer

**DOI:** 10.1038/s42003-024-06085-w

**Published:** 2024-04-01

**Authors:** Xiaoling Xu, Ding Wang, Weizhen Xu, Huihui Li, Ning Chen, Na Li, Qifeng Yao, Wei Chen, Jianxiang Zhong, Weimin Mao

**Affiliations:** 1 Department of Radiation Oncology, Shanghai Pulmonary Hospital, Tongji University School of Medicine, 507 Zhengmin Road, Shanghai, China; 2 Key laboratory on Diagnosis and Treatment Technology on Thoracic Cancer, Zhejiang Cancer Research Institute, 38 Guangji Road, Hangzhou, China; 3 The Second Clinical Medical College of Wenzhou Medical University, 268 West Xueyue Road, Wenzhou, China; 4 The Second Clinical Medical College of Zhejiang Chinese Medical University, 548 Binwen Road, Hangzhou, China; 5 School of Life Science and Technology, Southeast University, 2 Sipailou, Nanjing, China; 6 Department of Thoracic Oncology, Zhejiang Cancer Hospital, Institute of Basic Medicine and Cancer (IBMC), Chinese Academy of Sciences, 1 Banshan East Road, Hangzhou, China

**Keywords:** Non-small-cell lung cancer, DNA methylation

Correction to: *Communications Biology* 10.1038/s42003-024-05801-w, published online 20 February 2024

In this Article, there was a mistake in the order of the corresponding authors.

The author list was originally: Xiaoling Xu^1^, Ding Wang^2^, Weizhen Xu^2^, Huihui Li^3,2^, Ning Chen^4^, Na Li^3^, Qifeng Yao^3^, Jianxiang Zhong, Wei Chen^2^, Weimin Mao^2,3,6^

This has now been corrected to: Xiaoling Xu^1^, Ding Wang^2^, Weizhen Xu^2^, Huihui Li^3,2^, Ning Chen^4^, Na Li^3^, Qifeng Yao^3^, Wei Chen^2^, Jianxiang Zhong^5^, Weimin Mao^2,3,6^

