# Supplementary material for: NIPBL-mediated RAD21 facilitates tumorigenicity by the PI3K pathway in non-small-cell lung cancer
Source: Commun Biol. 2024 Feb 20;7:206. doi: 10.1038/s42003-024-05801-w (PMC10879132; doi:10.1038/s42003-024-05801-w)

Supplementary tables

Supplementary Table 1 Sequences of siRNAs

siRNAs		Sequences (5'-3')
**RAD21-595**	Sense	GCAGCUUAUAAUGCCAUUATT
	Antisense	UAAUGGCAUUAUAAGCUGCTT
**RAD21-677**	Sense	CCCAGCAGUUCAGCUUGAATT
	Antisense	UUCAAGCUGAACUGCUGGGTT
**RAD21-955**	Sense	GGAAAUGAUGGUGGAAUAUTT
	Antisense	AUAUUCCACCAUCAUUUCCTT
**siNIPBL2**	Sense	UGUUUUCUUUUUUUCAAAGGC
	Antisense	CUUUGAAAAAAAGAAAACAAG
**siNIPBL3**	Sense	UGCUUUUCUCCCUGAAAACAU
	Antisense	GUUUUCAGGGAGAAAAGCAUG
**siKDM6B1**	Sense	AAAGAAGAGCUCUUCUAAGAU
	Antisense	CUUAGAAGAGCUCUUCUUUGG
**siKDM6B2**	Sense	UCGAAACUUCCCUUUCACCUU
	Antisense	GGUGAAAGGGAAGUUUCGAGA

**siKDM6B3**	Sense	UGGAUUUGACGUUCUUCACCU
	Antisense	GUGAAGAACGUCAAAUCCAUC
**siEZH2 1**	Sense	GGAUGUGGAUACUCCUCCA
	Antisense	UGGAGGAGUAUCCACAUCC
**siEZH2 2**	Sense	UGUAGUUCAGAGUGUCAAA
	Antisense	UUUGACACUCUGAACUACA
**siEZH2 3**	Sense	GAAGUAAAGAGUAUGUUUA
	Antisense	UAAACAUACUCUUUACUUC
**siMETTL3**	Sense	CUGCAAGUAUGUUCACUAUGA
	Antisense	UCAUAGUGAACAUACUUGCAG
**siMETTL14**	Sense	GCUGGACUUGGGAUGAUAUUA
	Antisense	UAAUAUCAUCCCAAGUCCAGC
**siRNA NC**	Sense	UUCUCCGAACGUGUCACGU
	Antisense	ACGUGACACGUUCGGAGAA

Supplementary Table 2 Multivariate analysis between RAD21 expression and prognosis in NSCLC patients

Factors	Characteristics		Overall survival		
	Unfavorable	Favorable	Hazard ratio	95% CI	*P* value
**Family history of cancer**	Yes	No	0.445	0.166-1.192	0.107
**RAD21 expression**	Overexpression	Low expression	4.139	1.671-10.256	0.002
**P53 expression**	Yes	No	0.51	0.239-1.085	0.080

Supplementary Table 3 The correlation between clinicopathological features and RAD21 expression in NSCLC patients

Characteristics	Number of patients	RAD21 low expression (52 cases)	RAD21 overexpression (12 cases)	*P* value
**Gender**				
Female	22	20	2	0.193
Male	42	32	10	
**Age**				
<60 years old	34	28	6	0.810
≥60 years old	30	24	6	
**Smoking**				
Never	20	16	4	0.863
Ever	44	36	8	
**Alcohol consumption**				
Never	40	33	7	0.741
Ever	24	19	5	
**Family history of cancer**				
No	44	36	8	1.000
Yes	18	3	15	
**Pathological types**				
Squamous cell carcinomas	20	14	6	0.131
Adenocarcinoma	43	37	6	
**Differentiation**				
Intermediate or well	24	18	6	0.216

Poor	39	34	5	
**Primary tumor**				
T1	18	16	2	0.327
T2+T3+T4	46	36	10	
**Lymph node metastasis**				
N0	25	19	6	0.389
N1-2	39	33	6	
**Venous/lymphatic invasion**				
No	50	40	10	0.628
Yes	14	12	2	
**Clinical stage**				
I	37	30	7	0.968
II+III	27	22	5	
**Chemotherapy**				
No	36	12	24	0.469
Yes	28	7	21	
**Radiotherapy**				
No	52	42	10	1.000
Yes	12	10	2	
**P53 expression**				
Negative	31	28	3	0.067
Positive	19	13	6	

Supplementary Figures

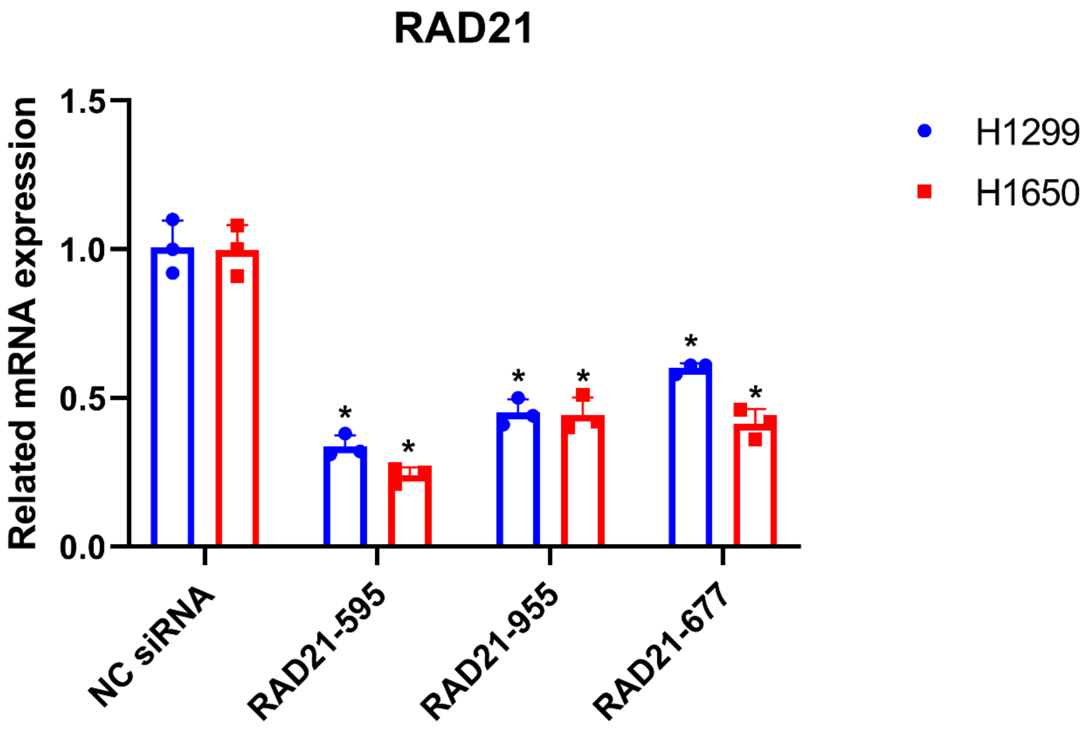

Supplementary Figure 1. RAD21 is downregulated by shRNAs. RAD21 mRNA level detected in H1299 and H1650 cells transfected with or without RAD21 shRNAs. Error bars indicate the standard error of the mean (SEM). N = 3. *P < 0.05.

Matrix ID	Name	Score	Relative score	Sequence ID	Start	End	Strand	Predicted sequence
MA0139.1	MA0139.1.CTCF	14.688376	0.8734353702347815	PI3K	962	980	+	agaccagtagggggagaaa
MA0139.1	MA0139.1.CTCF	9.677284	0.8152582496095275	PI3K	1259	1277	+	aggactgcagagggctgtg
MA0139.1	MA0139.1.CTCF	7.9587264	0.7953063623290838	PI3K	1967	1985	+	tagcgaggagagggagcga

Supplementary Figure 2. RAD21/CTCF binding site on human *PI3K* gene promoter. RAD21/CTCF binding sites on human *PI3K* gene promoter predicted using JASPAR website (https://jaspar.genereg.net/).

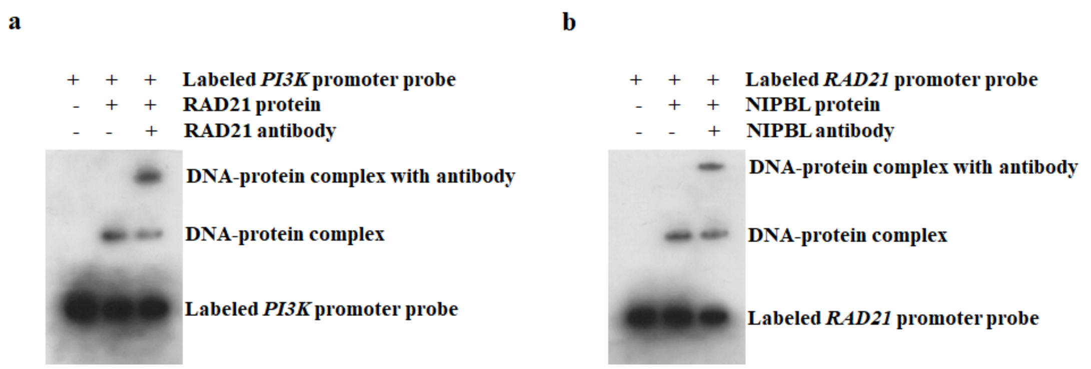

Supplementary Figure 3. The DNA binding ability of RAD21 and NIPBL *the DNA binding ability of RAD21 and NIPBL in vitro*. a The representative image of EMSA using a probe corresponding to *PI3K* gene promoter together with RAD21 protein and RAD21 antibody. **b** The representative image of EMSA using a probe corresponding to *RAD21* gene promoter together with NIPBL protein and NIPBL antibody.

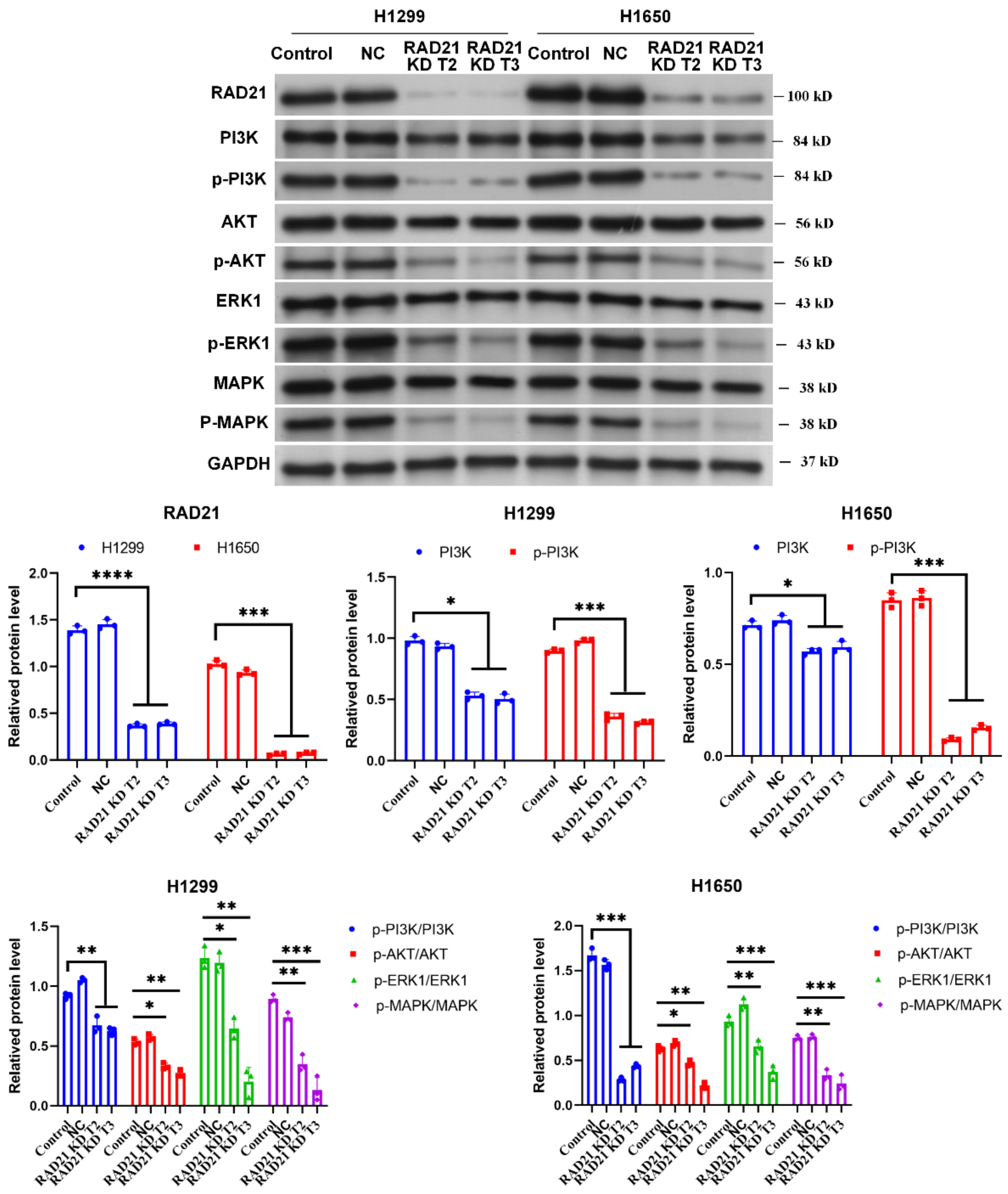

Supplementary Figure 4. Protein levels of RAD21 and factors involved in the PI3K pathway are decreased in RAD21-KD H1299 and H1650 cells. Protein levels of RAD21 and factors involved in the PI3K pathway detected in RAD21-KD H1299 and H1650 cells. The bar graphs showed the quantification of protein levels. Error bars indicate the standard error of the mean (SEM). N = 3. *P < 0.05, **P < 0.01, ***P < 0.001, ****P<0.0001.

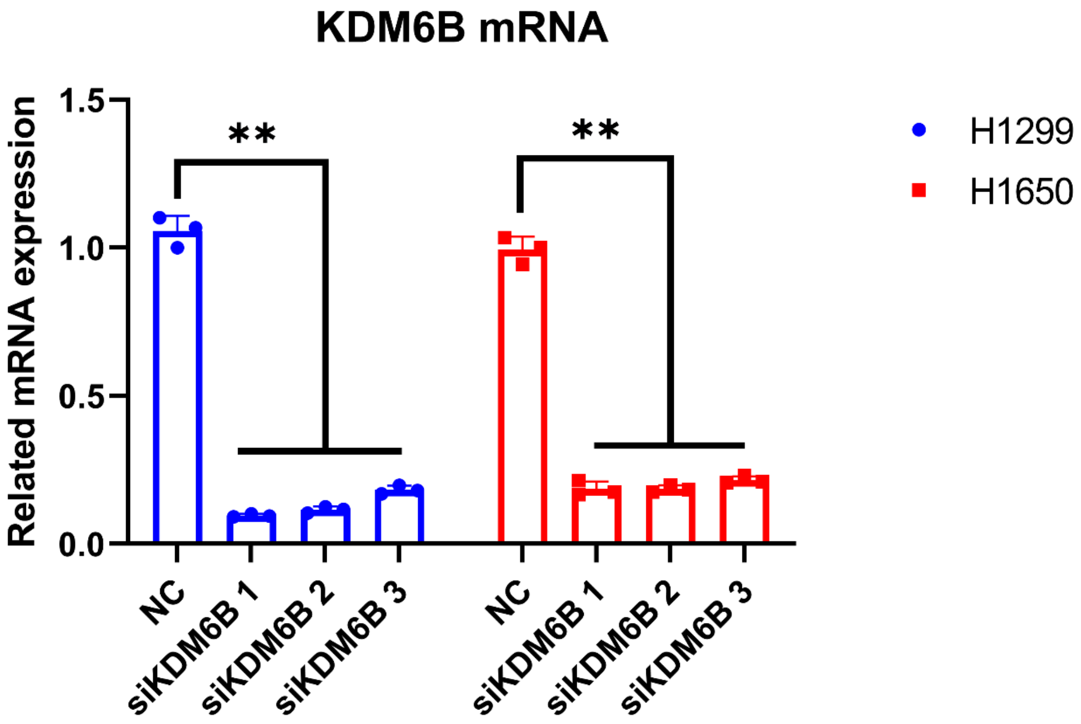

Supplementary Figure 5. KDM6B is downregulated by siRNAs. KDM6B mRNA level detected in H1299 and H1650 cells transfected with or without KDM6B siRNAs. Error bars indicate the standard error of the mean (SEM). N = 3. **$P < 0.01$.

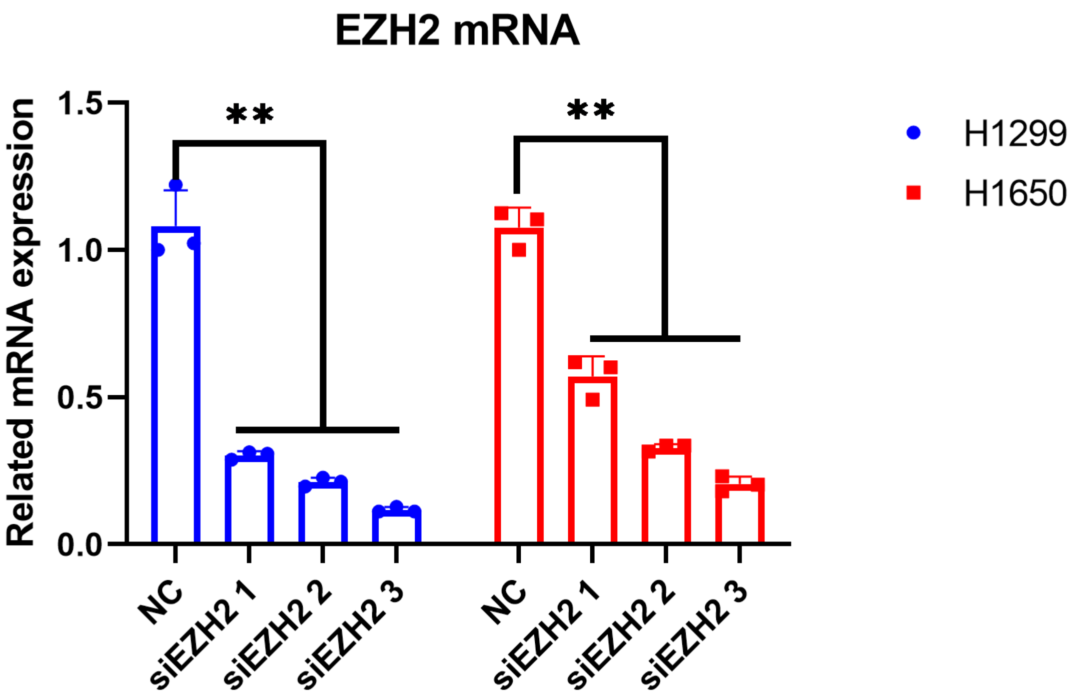

Supplementary Figure 6. EZH2 is downregulated by siRNAs. EZH2 mRNA level detected in H1299 and H1650 cells transfected with or without EZH2 siRNAs. Error bars indicate the standard error of the mean (SEM). N = 3. **$P < 0.01$.

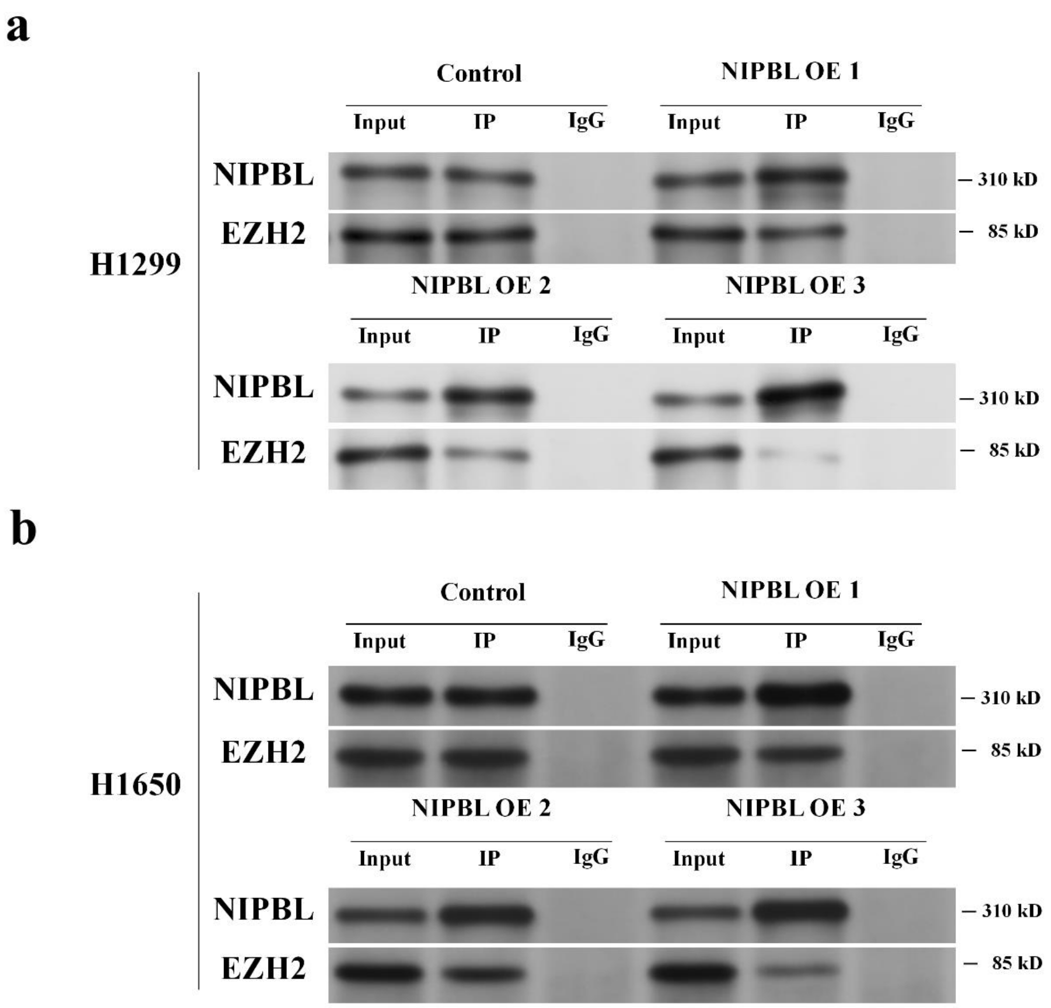

Supplementary Figure 7. NIPBL blocked the interaction of RAD21 and EZH2 through competitively binding with RAD21. Representative images of Co-IP using an anti-RAD21 antibody in scrambled and different dosages of NIPBL expression vector (0.5 μg, 1 μg, and 2 μg) transfected H1299 (**a**) and H1650 (**b**) cells. Rabbit IgG was used as a negative control. OE: overexpression.

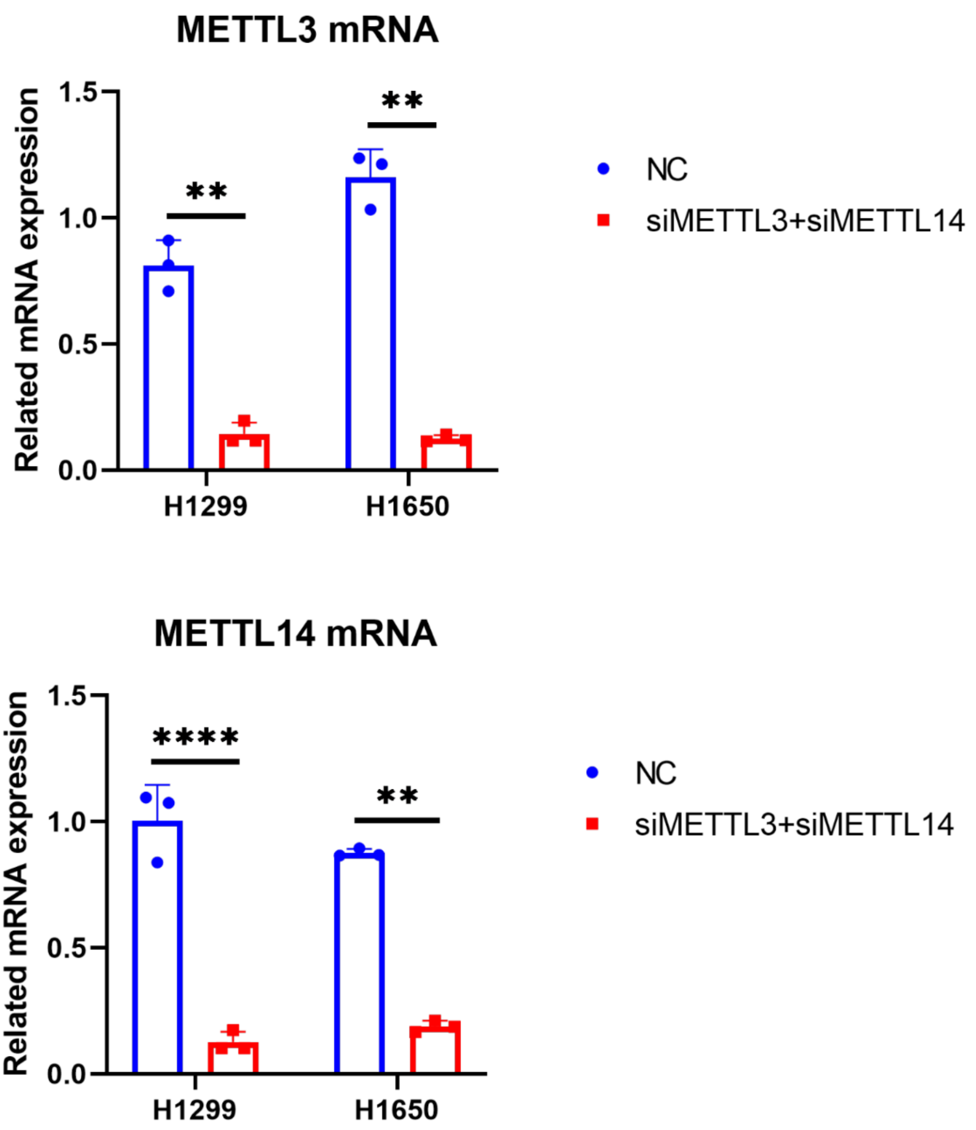

Supplementary Figure 8. METTL3 and METTL14 are downregulated by siRNAs. METTL3 and METTL14 mRNA level detected in H1299 and H1650 cells transfected with or without METTL3 and METTL14 siRNAs. Error bars indicate the standard error of the mean (SEM). N = 3. **P < 0.01, ****P<0.0001.

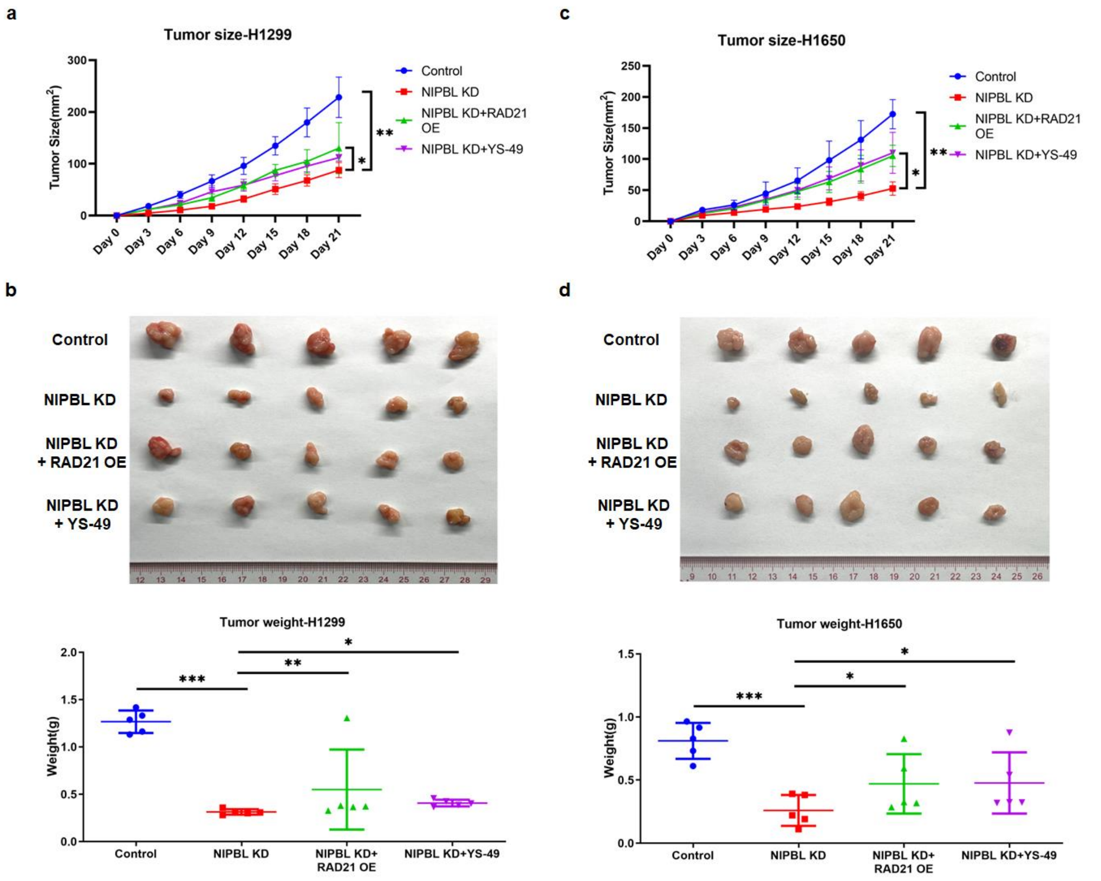

Supplementary Figure 9. NIPBL-mediated RAD21 accelerates the *in vivo* tumorigenicity of NSCLC via the PI3K pathway. a, c Quantification of the volume of xenograft tumors derived from control and NIPBL-KD plus RAD21-OE H1299 (A) and H1650 (C) cells in the presence and absence of YS-49. N = 5. **b, d** Images and quantification of xenograft tumors weight formed in nude mice injected with control and NIPBL-KD plus RAD21-OE H1299 (B) and H1650 (D) cells in the presence and absence of YS-49. Error bars indicate the standard error of the mean (SEM). N = 5. KD: knockdown; OE: overexpression. *P < 0.05, **P < 0.01, ***P<0.001.

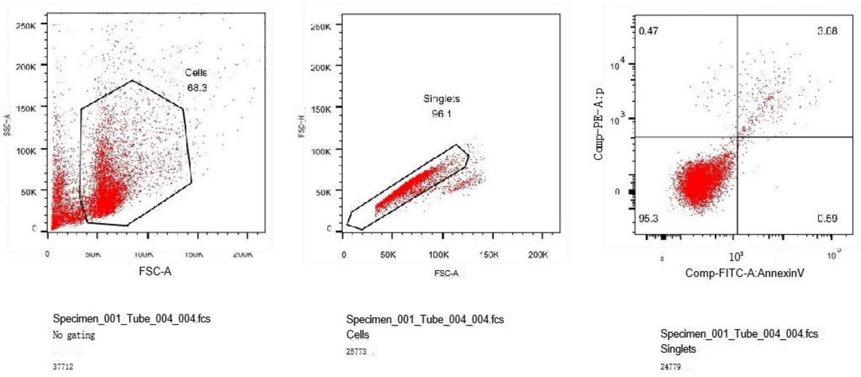

Supplementary Figure 10. Gating strategy for H1299 cells.

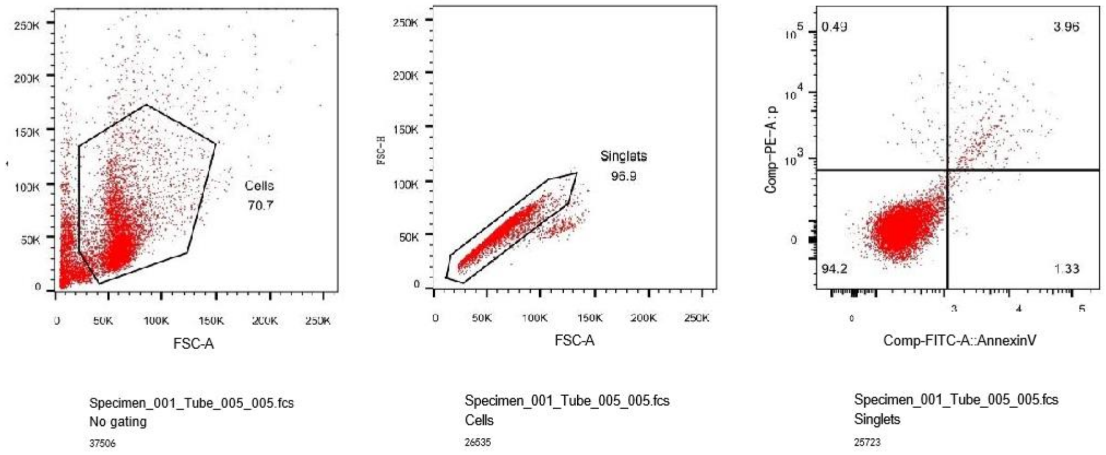

Supplementary Figure 11. Gating strategy for H1650 cells.

Uncropped and unedited blotgel images

Figure 1d

RAD21

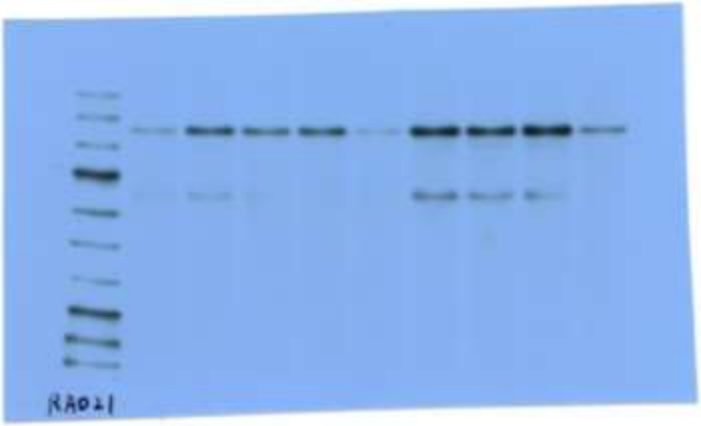

GAPDH

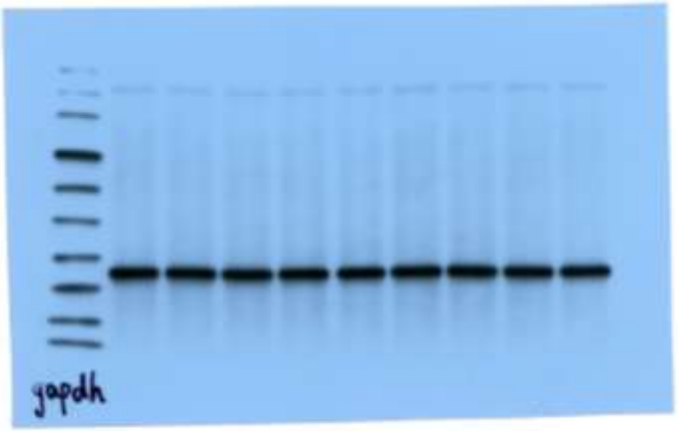

Figure 4d

PI3K

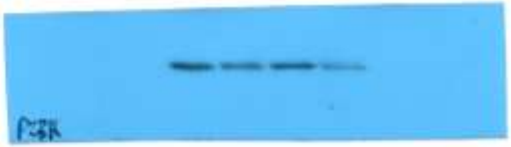

p-PI3K

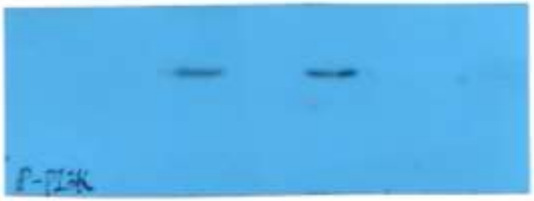

GAPDH

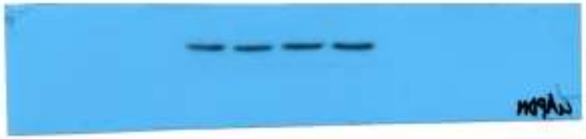

Figure 6a

H1299

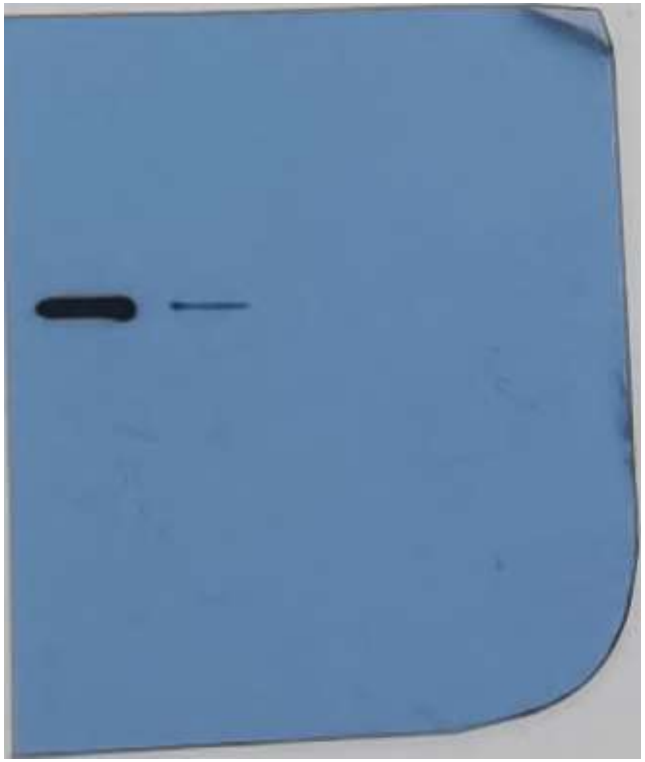

H1650

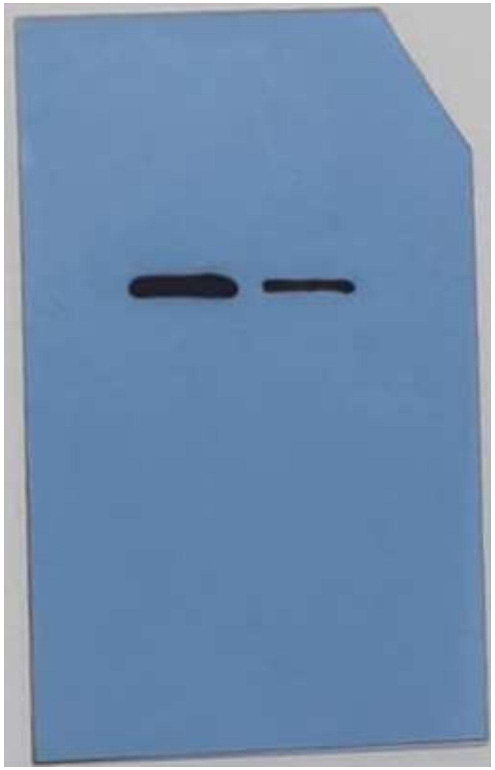

Figure 7a

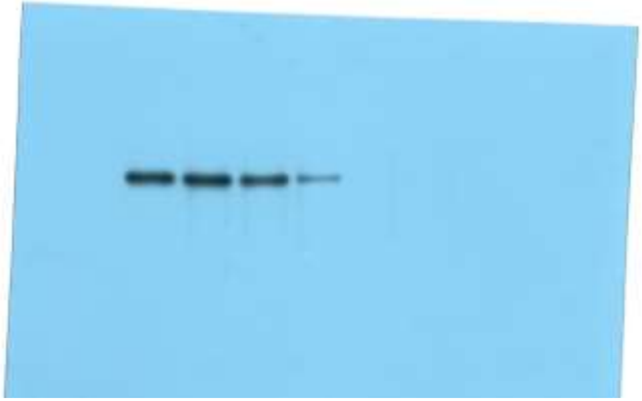

Figure 7c

H1299

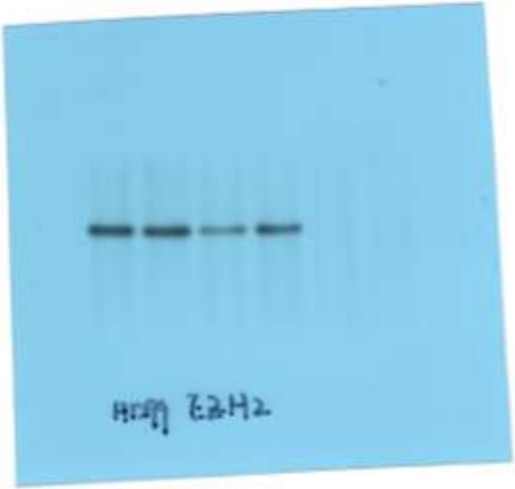

H1650

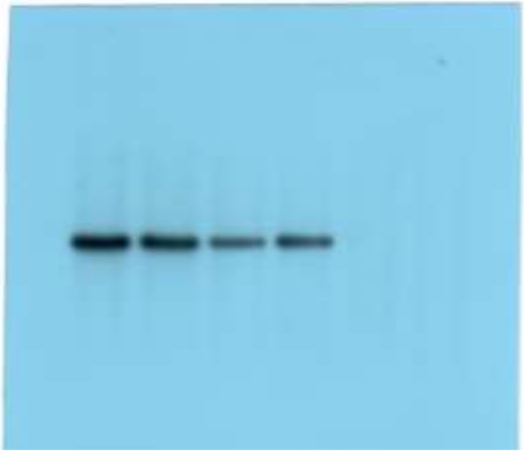

Supplementary Figure 3

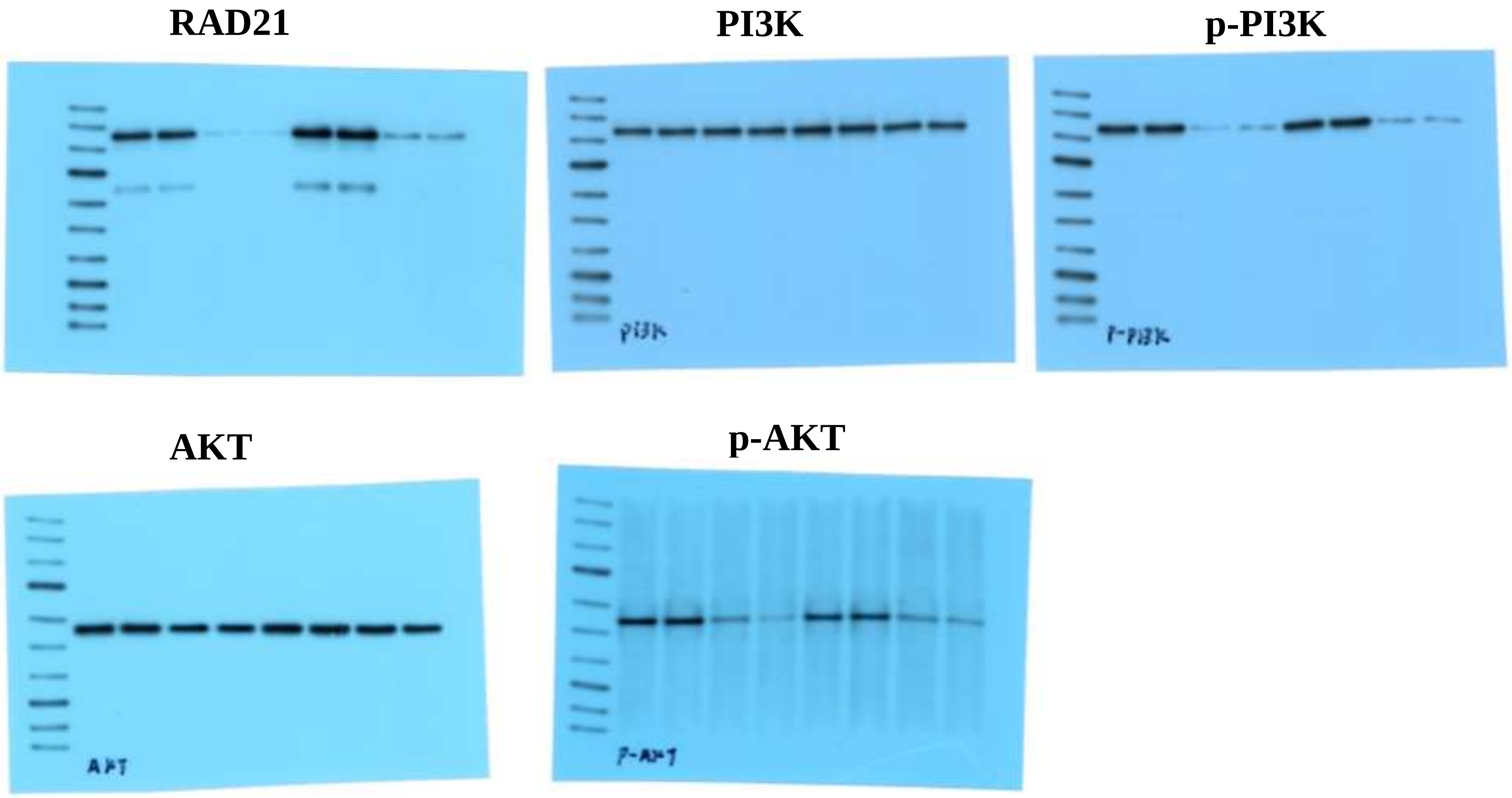

Supplementary Figure 3

ERK1

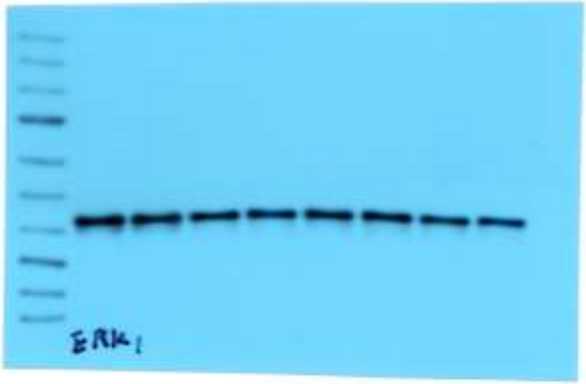

p-ERK1

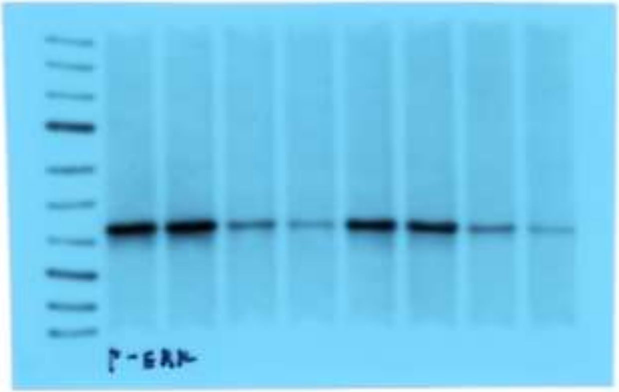

MAPK

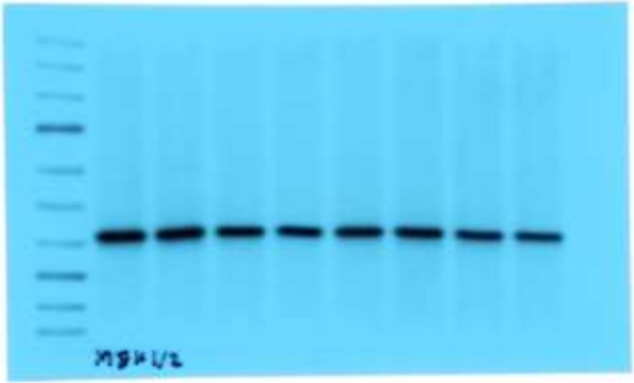

p-MAPK

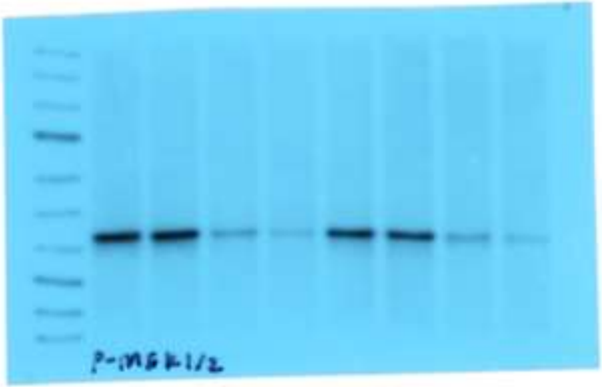

GAPDH

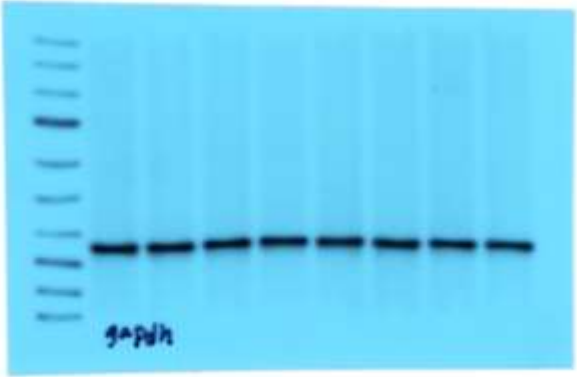

Supplementary Figure 7a

EZH2 **NIPBL**

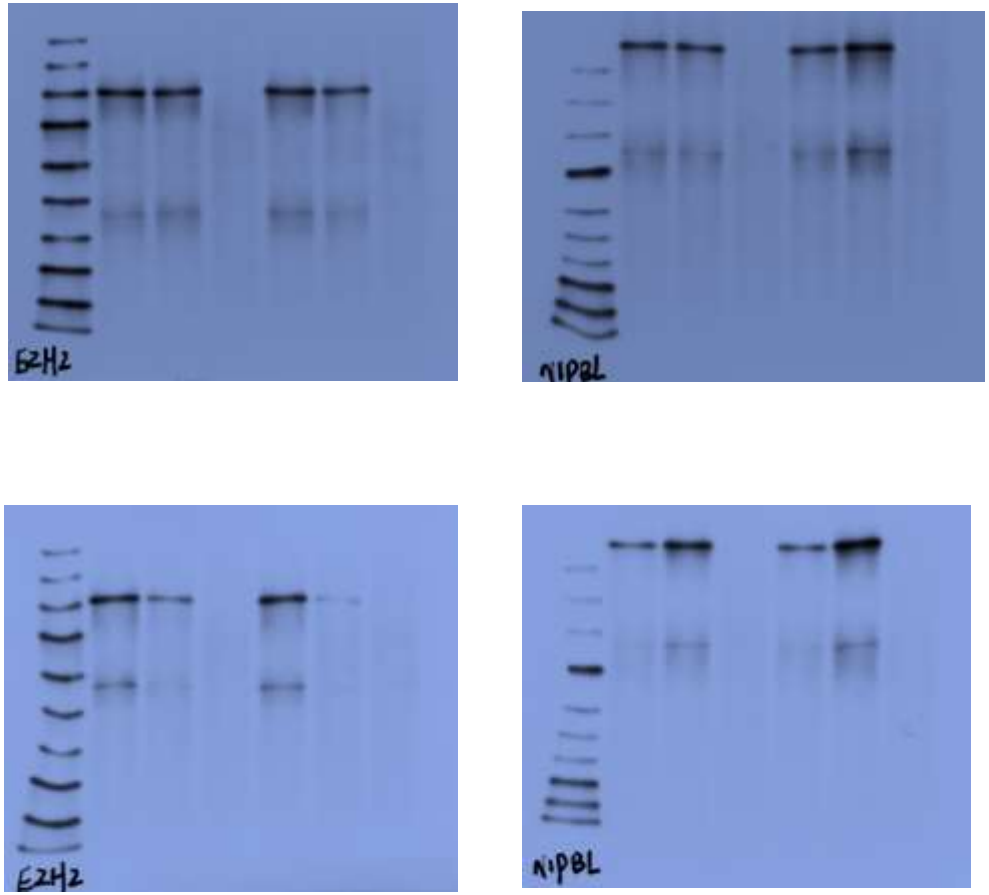

Supplementary Figure 7b

EZH2 | NIPBL

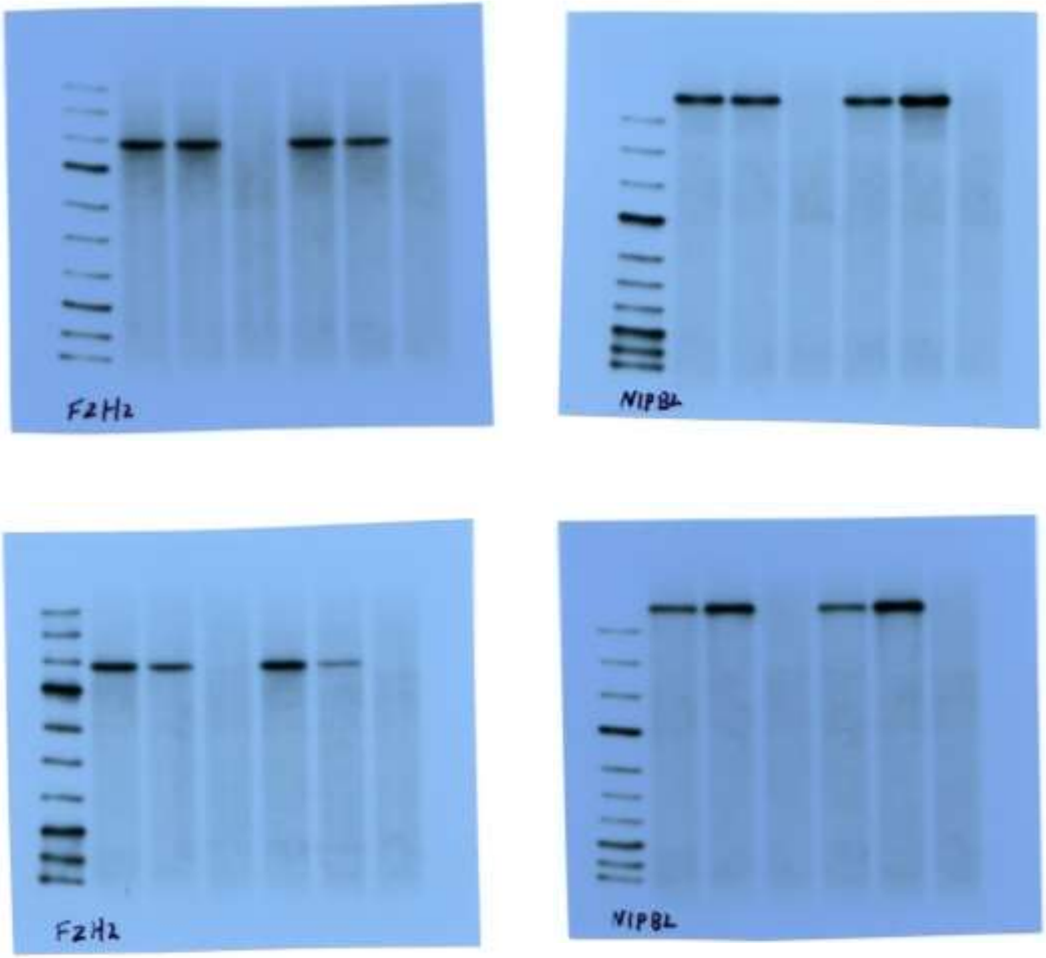

Supplement: Supplementary file 1 — Supplementary Information [file 42003_2024_5801_MOESM1_ESM.pdf]
