# Supplementary material for: NIPBL-mediated RAD21 facilitates tumorigenicity by the PI3K pathway in non-small-cell lung cancer
Source: Commun Biol. 2024 Feb 20;7:206. doi: 10.1038/s42003-024-05801-w (PMC10879132; doi:10.1038/s42003-024-05801-w)
Supplement: Supplementary file 4 — Reporting Summary [file 42003_2024_5801_MOESM4_ESM.pdf]

nature portfolio

Corresponding author(s): Weimin Mao

Last updated by author(s): Oct 10, 2023

Reporting Summary

Nature Portfolio wishes to improve the reproducibility of the work that we publish. This form provides structure for consistency and transparency in reporting. For further information on Nature Portfolio policies, see our Editorial Policies and the Editorial Policy Checklist.

Statistics

For all statistical analyses, confirm that the following items are present in the figure legend, table legend, main text, or Methods section.

n/a	Confirmed	
☐	☒	The exact sample size (*n*) for each experimental group/condition, given as a discrete number and unit of measurement
☐	☒	A statement on whether measurements were taken from distinct samples or whether the same sample was measured repeatedly
☐	☒	The statistical test(s) used AND whether they are one- or two-sided<br>*Only common tests should be described solely by name; describe more complex techniques in the Methods section.*
☒	☐	A description of all covariates tested
☒	☐	A description of any assumptions or corrections, such as tests of normality and adjustment for multiple comparisons
☐	☒	A full description of the statistical parameters including central tendency (e.g. means) or other basic estimates (e.g. regression coefficient) AND variation (e.g. standard deviation) or associated estimates of uncertainty (e.g. confidence intervals)
☐	☒	For null hypothesis testing, the test statistic (e.g. *F*, *t*, *r*) with confidence intervals, effect sizes, degrees of freedom and *P* value noted<br>*Give P values as exact values whenever suitable.*
☒	☐	For Bayesian analysis, information on the choice of priors and Markov chain Monte Carlo settings
☒	☐	For hierarchical and complex designs, identification of the appropriate level for tests and full reporting of outcomes
☐	☒	Estimates of effect sizes (e.g. Cohen's *d*, Pearson's *r*), indicating how they were calculated

Our web collection on statistics for biologists contains articles on many of the points above.

Software and code

Policy information about availability of computer code

Data collection	No software was used.
Data analysis	No software was used.

For manuscripts utilizing custom algorithms or software that are central to the research but not yet described in published literature, software must be made available to editors and reviewers. We strongly encourage code deposition in a community repository (e.g. GitHub). See the Nature Portfolio guidelines for submitting code & software for further information.

Data

Policy information about availability of data

All manuscripts must include a data availability statement. This statement should provide the following information, where applicable:

- Accession codes, unique identifiers, or web links for publicly available datasets
- A description of any restrictions on data availability
- For clinical datasets or third party data, please ensure that the statement adheres to our policy

All data generated and analyzed during this study are included in this manuscript.

Research involving human participants, their data, or biological material

Policy information about studies with human participants or human data. See also policy information about sex, gender (identity/presentation), and sexual orientation and race, ethnicity and racism.

Reporting on sex and gender	Information on sex and gender has been presented in Table 2.
Reporting on race, ethnicity, or other socially relevant groupings	Information on race, ethnicity or other socially relevant groupings has not been collected.
Population characteristics	Population characteristics has been described in Table 2.
Recruitment	How pariticipants were recruited has been described in "Materiials and Methods".
Ethics oversight	The name(s) of the board and institution that approved the study prptocol has been disclosed in this manuscript.

Note that full information on the approval of the study protocol must also be provided in the manuscript.

Field-specific reporting

Please select the one below that is the best fit for your research. If you are not sure, read the appropriate sections before making your selection.

☒ Life sciences ☐ Behavioural & social sciences ☐ Ecological, evolutionary & environmental sciences

For a reference copy of the document with all sections, see nature.com/documents/nr-reporting-summary-flat.pdf

Life sciences study design

All studies must disclose on these points even when the disclosure is negative.

Sample size	Sample size has been disclosed in this manuscript.
Data exclusions	Data exclusions has been disclosed in this manuscript.
Replication	Replication has been disclosed in this manuscript.
Randomization	Randomization has been disclosed in this manuscript.
Blinding	Blinding has been disclosed in this manuscript.

Reporting for specific materials, systems and methods

We require information from authors about some types of materials, experimental systems and methods used in many studies. Here, indicate whether each material, system or method listed is relevant to your study. If you are not sure if a list item applies to your research, read the appropriate section before selecting a response.

Materials & experimental systems

n/a	Involved in the study	
☐	☒	Antibodies
☐	☒	Eukaryotic cell lines
☒	☐	Palaeontology and archaeology
☐	☒	Animals and other organisms
☒	☐	Clinical data
☒	☐	Dual use research of concern
☒	☐	Plants

Methods

n/a	Involved in the study	
☒	☐	ChIP-seq
☐	☒	Flow cytometry
☒	☐	MRI-based neuroimaging

Antibodies

Antibodies used	Informaiton of antibodies has been described in this manuscript.
Validation	Validation of antibodies has been described in this manuscript.

Eukaryotic cell lines

Policy information about cell lines and Sex and Gender in Research

Cell line source(s)	Cell line source(s) has been stated in this manuscript.
Authentication	Authentication procedures have been stated in this manuscript.
Mycoplasma contamination	All cell lines are negative to mycoplasma contamination.
Commonly misidentified lines (See ICLAC register)	Commonly misidentified lines have been used in this manuscript.

Animals and other research organisms

Policy information about studies involving animals; ARRIVE guidelines recommended for reporting animal research, and Sex and Gender in Research

Laboratory animals	Informaiton of laboratory animals has been stated in "Materials and methods" section.
Wild animals	This study did not involve wild animals.
Reporting on sex	Sex of laboratory animals has been stated in "Materials and methods" section.
Field-collected samples	Filed-collected samples have been described in "Materials and methods" section.
Ethics oversight	The name(s) of board and institution that approved the study protocol has been stated in this manucript.

Note that full information on the approval of the study protocol must also be provided in the manuscript.

Plants

Seed stocks	This study did not involve plants.
Novel plant genotypes	This study did not involve platns.
Authentication	This study did not involve plants.

Flow Cytometry

Plots

Confirm that:

☒ The axis labels state the marker and fluorochrome used (e.g. CD4-FITC).

☒ The axis scales are clearly visible. Include numbers along axes only for bottom left plot of group (a 'group' is an analysis of identical markers).

☒ All plots are contour plots with outliers or pseudocolor plots.

☒ A numerical value for number of cells or percentage (with statistics) is provided.

Methodology

Sample preparation	Sample preparation has been described in "Materials and methods" section.
Instrument	Instrucment has been described in "Materials and methods" section.
Software	Software has been dedcribed in "Materials and methods" section.
Cell population abundance	Cell population abundance has been described in "Matherials and methods" section.

Gating strategy | Gating strategy has been described in "Matherials and methods" section.

☒ Tick this box to confirm that a figure exemplifying the gating strategy is provided in the Supplementary Information.
